# Phylogenetic Reconstruction of the Calosphaeriales and Togniniales Using Five Genes and Predicted RNA Secondary Structures of ITS, and *Flabellascus tenuirostris* gen. et sp. nov.

**DOI:** 10.1371/journal.pone.0144616

**Published:** 2015-12-23

**Authors:** Martina Réblová, Walter M. Jaklitsch, Kamila Réblová, Václav Štěpánek

**Affiliations:** 1 Department of Taxonomy, Institute of Botany of the Academy of Sciences of the Czech Republic, Průhonice, Czech Republic; 2 Department of Forest and Soil Sciences, Forest Pathology and Forest Protection, Institute of Forest Entomology, BOKU-University of Natural Resources and Life Sciences, Vienna, Austria; 3 Department of Botany and Biodiversity Research, Division of Systematic and Evolutionary Botany, University of Vienna, Vienna, Austria; 4 Faculty of Medicine, Masaryk University, Brno, Czech Republic; 5 Central European Institute of Technology, Masaryk University, Brno, Czech Republic; 6 Laboratory of Enzyme Technology, Institute of Microbiology of the Academy of Sciences of the Czech Republic, Prague, Czech Republic; University of Szeged, HUNGARY

## Abstract

The Calosphaeriales is revisited with new collection data, living cultures, morphological studies of ascoma centrum, secondary structures of the internal transcribed spacer (ITS) rDNA and phylogeny based on novel DNA sequences of five nuclear ribosomal and protein-coding loci. Morphological features, molecular evidence and information from predicted RNA secondary structures of ITS converged upon robust phylogenies of the Calosphaeriales and Togniniales. The current concept of the Calosphaeriales includes the Calosphaeriaceae and Pleurostomataceae encompassing five monophyletic genera, *Calosphaeria*, *Flabellascus* gen. nov., *Jattaea*, *Pleurostoma* and *Togniniella*, strongly supported by Bayesian and Maximum Likelihood methods. The structural elements of ITS1 form characteristic patterns that are phylogenetically conserved, corroborate observations based on morphology and have a high predictive value at the generic level. Three major clades containing 44 species of *Phaeoacremonium* were recovered in the closely related Togniniales based on ITS, actin and β-tubulin sequences. They are newly characterized by sexual and RNA structural characters and ecology. This approach is a first step towards understanding of the molecular systematics of *Phaeoacremonium* and possibly its new classification. In the Calosphaeriales, *Jattaea aphanospora* sp. nov. and *J*. *ribicola* sp. nov. are introduced, *Calosphaeria taediosa* is combined in *Jattaea* and epitypified. The sexual morph of *Phaeoacremonium cinereum* was encountered for the first time on decaying wood and obtained *in vitro*. In order to achieve a single nomenclature, the genera of asexual morphs linked with the Calosphaeriales are transferred to synonymy of their sexual morphs following the principle of priority, i.e. *Calosphaeriophora* to *Calosphaeria*, *Phaeocrella* to *Togniniella* and *Pleurostomophora* to *Pleurostoma*. Three new combinations are proposed, i.e. *Pleurostoma ochraceum* comb. nov., *P*. *repens* comb. nov. and *P*. *richardsiae* comb. nov. The morphology-based key is provided to facilitate identification of genera accepted in the Calosphaeriales.

## Introduction

The Calosphaeriales (Sordariomycetes) traditionally comprise wood-inhabiting perithecial ascomycetes that occupy specialized habitats between wood and periderm. The current concept includes two families, the Calosphaeriaceae encompassing *Calosphaeria* Tul. & C. Tul., *Jattaea* Berl. and *Togniniella* Réblová et al., and the monotypic Pleurostomataceae [1–8]. These genera are characterized by non-stromatic, rarely stromatic perithecia with an eccentric papilla or long central neck, asci with a conspicuously thickened apex lacking a visible discharge mechanism and containing hyaline, allantoid to suballantoid, oblong or subcylindrical, septate or non-septate ascospores, and apically free, septate paraphyses. The calosphaeriaceous ascoma centrum is unique in the Sordariomycetes; it comprises persistent, sparsely branched, short or elongated ascogenous hyphae with minute cells formed from croziers in sympodial succession, asci in predominantly spicate arrangement and persistent paraphyses. The centrum is a hallmark of the order and the main character that led Munk [1] to describe the Calosphaeriaceae.

Members of the Calosphaeriales are macroscopically inconspicuous and frequently found when searching for other fungi beneath the periderm, especially stromatic ascomycetes of the Diaporthales and Diatrypaceae. Although the majority of calosphaeriaceous fungi are saprobic or hypersaprobic found in company with old stromata and ascomata of other ascomycetes on woody plants, recently some species have been isolated from wood of fruit trees showing canker symptoms, viz. *Calosphaeria africana* Damm & Crous, *C*. *pulchella* (Pers.) J. Schröt. and *Jattaea algeriensis* Berl. [7, 9], and three species of *Pleurostomophora* Vijaykr. et al. were described as opportunistic human pathogens causing subcutaneous phaeohyphomycosis including formation of cysts or true mycetoma [10–13].

The asexual morphs linked to the Calosphaeriales comprise reduced, morphologically similar dematiaceous hyphomycetes with phialidic conidiogenesis similar to *Phialophora* Medlar and *Phaeoacremonium* W. Gams, Crous & M.J. Wingf. They are characterized by hyaline, ellipsoid, oblong, reniform, allantoid to suballantoid conidia borne on a single locus on monophialidic conidiogenous cells. The conidiophores are semi-macronematous, hyaline to translucent pale brown or yellow-brown, sometimes branched, often reduced to conidiogenous cells. These asexual morphs were discovered recently based on cultivation experiments and three new genera were introduced, viz. *Calosphaeriophora* Réblová et al. and *Phaeocrella* Réblová et al. as asexual morphs of *Calosphaeria* and *Togniniella* [4], and *Pleurostomophora* was linked to *Pleurostoma* Tul. & C. Tul. [5]. The described asexual morphs of *Jattaea* have always been referred to as a *Phialophora*-like [7, 8].

Calosphaeriales are closely related to the Togniniales, which comprise only the family Togniniaceae introduced for *Phaeoacremonium* and its *Togninia* Berl. sexual morph by Réblová et al. [4]. The link between the two morphs was confirmed by mating experiments in culture and DNA sequence data by Mostert et al. [14]. *Phaeoacremonium* has been monographed [6] and several new species were introduced recently [15–21]. A phylogenetic analysis of β-tubulin and actin DNA sequences provided a statistically robust phylogeny of *Phaeoacremonium* and 43 analysed species were separated into three major clades by Maximum Parsimony method [18]. Although the majority of *Phaeoacremonium* species have been isolated from wood of *Vitis vinifera* L. showing symptoms of esca and Petri disease, *Prunus* spp., *Olea europea* L., other fruit and deciduous trees, occasionally from soil or insects, eleven species were reported to cause phaeohyphomycosis in humans [10–12, 22–27]. Members of the Calosphaeriales and Togniniales share non-stromatic, papillate or long-necked perithecial ascomata, hyaline ascospores, minute asci with a thickened apex lacking a visible discharge mechanism arranged in spicate clusters and a phialidic conidiogenesis. Morphologically *Phaeoacremonium* differs from the calosphaeriaceous fungi by non-stipitate asci and lack of a typical centrum comprising ascogenous hyphae with discrete, minute cells. Some of the sexual morphs of *Phaeoacremonium* were previously accommodated in *Calosphaeria* [6, 14, 28, 29].

During a survey of lignicolous ascomycetes in Austria, the Czech Republic and France, we encountered three undescribed calosphaeriaceous fungi. Six collections of an unknown perithecial ascomycete strongly resembling *Togniniella* were collected on decaying wood of *Fagus sylvatica* L. and *Quercus cerris* L. Although no conidiophores were formed on the host, cultures derived from ascospore isolates yielded identical asexual morphs with two types of hyaline, non-septate conidia formed on monophialides arranged in whorls on dark brown conidiophores. Two other collections made on twigs of *Ribes petraeum* Wulfen in Austria and a specimen on a branch of *Crataegus* sp. in France represent two undescribed species of *Jattaea*. These specimens were successfully isolated into axenic culture and yielded a reduced phialidic hyphomycete similar to *Phialophora* producing hyaline to pale brown mycelium and hyaline, non-septate, oblong-ellipsoidal, slightly curved conidia on monophialidic conidiogenous cells *in vitro*.

Recent collections of *Calosphaeria taediosa* Sacc., *Jattaea aurea* Réblová & J. Fourn. and *J*. *tumidula* (Sacc.) Réblová were isolated into axenic culture for the first time. Their phialidic asexual morphs are described and illustrated in this study. *Calosphaeria taediosa* is remarkably similar to *Calosphaeria cryptospora* Munk [1] in 3–4-septate subcylindrical ascospores producing numerous ascoconidia within the asci and its frequent association with ascomata of *Cryptospora suffusa* (Fr.) Tul. & C. Tul. on *Alnus* spp. wood.

A fresh collection of a sexual morph of *Phaeoacremonium cinereum* D. Gramaje et al. [18] was made on decaying deciduous wood in southern France. In culture it yielded fertile ascomata and conidia, conidiogenous cells and conidiophores identical to those described in the protologue. *Phaeoacremonium cinereum* is a plant pathogen, known so far from two isolates from *Vitis vinifera* from Iran and Spain. The sexual morph, encountered for the first time, is described and illustrated below.

The main aim of this study is to revise the classification of the Calosphaeriales with additional collections and novel rDNA sequence data and characterise the three major clades distinguished in the Togniniales with the aid of molecular data, RNA structural data of ITS, sexual characters and ecology. We investigated phylogenetic relationships of the three undescribed fungi, and also of *C*. *taediosa*, *J*. *aurea* and *J*. *tumidula* with other members of the Calosphaeriales and compared asexual morphs linked to this order. The ascoma centrum is investigated and compared among members of the Calosphaeriales and Togniniales. We aimed to epitypify *Calosphaeria taediosa* and *J*. *tumidula* using recently collected material in Austria and France and a living culture of the latter species to provide morphological and molecular characterization of the epitype specimens. The alignment of homologous nucleotides of ITS sequences of strains of the unknown fungi with species of the Calosphaeriales and Togniniales has been hampered by significant differences in their length. Therefore, we performed a computational analysis of the RNA secondary (2D) structure of ITS in order to reveal some characteristic architectural elements [30–32], and verify a presumed existence of 2D models of ITS that may be genus-specific. The study of variability at the RNA structural level within each morphologically and phylogenetically defined group of species of the Calosphaeriales and Togniniales entailed a comparative analysis of predicted RNA secondary structures of ITS1 and ITS2. For phylogenetic analyses we utilised molecular sequence characters from the nuclear rDNA internal transcribed spacer barcode (ITS1-5.8S-ITS2), two ribosomal and two protein-coding loci. A key and list of genera accepted in the Calosphaeriales is presented.

## Material and Methods

### Morphological characterization of fungal strains and herbarium material

Dry ascomata were rehydrated with water; material was examined with an Olympus SZX12 dissecting microscope and centrum material (including asci, ascospores and paraphyses) was mounted in Melzer’s reagent, 90% lactic acid, lactophenol with cotton blue or aqueous cotton-blue (1 mg/ml). Hand sections of the ascomatal wall were studied in 3% KOH. All measurements were made in Melzer’s reagent. Means ± standard deviations (SD) based on 20–25 measurements are given for dimensions of asci, ascospores, conidia and conidiogenous cells. The terminology of phialide types of species of *Phaeoacremonium* was used according to Mostert et al. [6]. Images were captured by differential interference (DIC) or phase contrast (PC) microscopy using an Olympus DP70 camera operated by Imaging Software Cell on an Olympus BX51 compound microscope.

Multi-ascospore isolates were obtained from fresh material with the aid of a spore isolator (Meopta, Prague, Czech Republic). Isolates were grown on Modified Leonian’s agar (MLA) [33] and potato-carrot agar (PCA) [34]. Colonies were examined after 7, 14, 21 and 30 d incubated at 25°C in the dark. Living cultures are maintained at CBS-KNAW Fungal Biodiversity Centre, Utrecht, the Netherlands (CBS). Type and other herbarium material is deposited in the Mycological Herbarium in the National Museum in Prague, Czech Republic (PRM) and in the Herbarium of the Institute of Botany, University of Vienna, Austria (WU). The ‘Online auction color chart’ [35] was used as the colour standard.

### DNA isolation, amplification and sequence alignment

Cultures used for DNA isolations were grown and procedures for amplifying and sequencing the internal transcribed spacer rDNA (ITS rDNA), small and large subunit nuclear ribosomal DNA (nuc18S rDNA, nuc28S rDNA), second largest subunit of RNA polymerase II (*rpb2*) were performed as described in [36]. Total nucleic acids were extracted from mycelia following the protocols of [37]. A fragment of the β-tubulin gene region was amplified and sequenced using the primers Bt2a/benA1 and Bt2b [38, 39]. Sequences were edited using Sequencher 5.0 software (Gene Codes Corp., Ann Arbor, MI, USA).

GenBank accession numbers for ITS, nuc28S, nuc18S, actin, β-tubulin and *rpb2* sequences determined for this study and other homologous sequences of members of the Calosphaeriales and Togniniales retrieved from GenBank and the CBS-KNAW strain collection are listed in S1 Table. Sequences were manually aligned in BioEdit v.7.1.9 [40]. The nuc18S and nuc28S alignments were enhanced by utilising the homologous 2D structure of *Saccharomyces cerevisiae* Meyen ex E.C. Hansen [41, 42] in order to improve the decisions on homologous characters and introduction of gaps. These procedures and alignment of the three protein-coding genes were performed as described in [43]. Predicted 2D models obtained for the ITS1 and ITS2 were used to determine the positions of homologous nucleotides in the ITS.

The single-locus data sets were examined for topological incongruence among loci (ITS: 82 sequences and 662 characters, nuc18S: 23 sequences and 1776 characters, nuc28S: 35 sequences and 1980 characters, actin: 46 sequences and 295 characters, β-tubulin: 71 sequences and 890 characters, *rpb2*: 11 sequences and 1117 characters). For individual loci, 500 bootstrap replicates were generated with RAxML-HPC v.7.0.3 [44, 45] and compared visually for topological conflicts among supported clades in phylogenetic trees. A conflict between two loci was assumed to occur when a clade appeared monophyletic with bootstrap support of ≥ 75% in one tree, but was not supported as monophyletic in another [46]. Individual, conflict-free alignments were concatenated to combine sequences for two subsequent phylogenetic analyses. The multiple sequence alignments are deposited in TreeBASE (Study no. 18161).

### Phylogenetic analyses

We performed two phylogenetic analyses. Phylogenetic relationships among members of the Calosphaeriales were resolved based on analysis of ITS, β-tubulin, nuc18S, nuc28S, and *rpb2* sequences of 37 isolates representing 21 species and five genera. *Phaeoacremonium fraxinopennsylvanicum* (T.E. Hinds) D. Gramaje, L. Mostert & Crous, *P*. *minimum* (Tul. & C. Tul) D. Gramaje, L. Mostert & Crous and *P*. *novae-zealandiae* L. Mostert, W. Gams & Crous were used to root the tree. In the second analysis we investigated relationships among 44 *Phaeoacremonium* species with the combined ITS, actin and β-tubulin sequences. *Wuestneia molokaiensis* Crous & J.D. Rogers and *Gnomonia gnomon* (Tode) J. Schröt. were used to root the tree.

We analysed the 5’ half of the nuc28S, the entire nuc18S, ITS, the 5–7 segments of the *rpb2* and coding and non-coding regions of β-tubulin (exons 2, 3, 4, 5 and partial 6) and actin (exons 1, 2 and partial 3). 145 bases of the nuc18S and 654 of the nuc28S were excluded from the analyses because of the incompleteness of the 5’- and 3’-ends of the majority of the available sequences. The combined data set was partitioned into ITS, nuc28S, nuc18S, *rpb2*, and coding and non-coding regions of actin and β-tubulin.

Maximum likelihood (ML) and Bayesian inference (BI) analyses were used to estimate phylogenetic relationships. ML analysis was performed with RAxML-HPC v.7.0.3 [44, 45] with a GTRCAT model of evolution. Nodal support was determined by non-parametric bootstrapping (BS) with 1 000 replicates. BI analysis was performed in a likelihood framework as implemented in the MrBayes v.3.0b4 software package to reconstruct phylogenetic trees [47]. Initially, an appropriate DNA substitution model that would best fit the model of DNA evolution for each sequence data set and each partition of the combined data sets was selected using MrModeltest2 v.2.3 [48]. Among the 24 models tested, the GTR+I+G substitution model was selected for the ITS, β-tubulin, nuc28S, *rpb2*; SYM+I for the coding region of actin, HKY+I+G for the non-coding region of actin and nuc18S. Multiple Bayesian searches using Metropolis-coupled Markov chain Monte Carlo sampling were conducted. One cold and three heated Markov chains were used in the analysis. Analyses were run for 10 million generations, with trees sampled every 1 000 generations. We used the Tracer v.1.6.0. [49] for analysis of trace files from Bayesian MCMC runs to assess whether we have run the analysis long enough to reach convergence. In both BI analyses the runs were long enough to effectively sample each distribution and to reach convergence. The first 50 000 trees, which represented the burn-in phase of the analysis, were discarded. The remaining trees were used for calculating posterior probabilities (PP) of recovered branches in the 50% majority rule consensus tree [50].

### Prediction of RNA secondary structure models of ITS1 and ITS2

Knowledge of 2D structure is essential for constructing a reliable multiple sequence alignment to compare nucleotides at homologous positions (in helices and loops) while searching for non-conserved co-evolving nucleotides that maintain base pairing. Consensus 2D structure models for the ITS1 and ITS2 were built using the PPfold program v.3.0 [51], which uses an explicit evolutionary model and a probabilistic model of structures and relies on multiple sequence alignment of related RNA sequences. Final 2D models created for all members of the Calosphaeriales were further improved using the Mfold program [52] and then adjusted manually if necessary, based on comparison of homologous positions in the alignment. The predicted 2D RNA structures of ITS1 and ITS2 were obtained in a dot bracket notation and were visualised and drawn using VARNA: Visualization Applet for RNA program [53].

To evaluate 2D RNA structures more precisely we classified the topology of three-way junction (family A, B or C) occurring in the determined ITS1 model and also predicted coaxial helical stacking arrangement in the junction. The freely available programs Junction Explorer [54] and Cartaj [55] were used for that purpose. These programs consider mainly length of the loop between helices, sequence content and either free-energy associated to base stacking interactions between the base pairs at the end of helices or frequency of the closing base pair types.

Computational analysis of the 2D RNA structure of ITS was based on 37 sequences representing 21 species of the Calosphaeriales and 45 sequences of 41 *Phaeoacremonium* species. Of the 46 *Phaeoacremonium* species described to date, five do not have their ITS sequences available, viz. *P*. *amygdalinum* D. Gramaje, Armengol & L. Mostert, *P*. *inconspicuum* (Rehm) D. Gramaje, L. Mostert & Crous, *P*. *krajdenii* L. Mostert, Summerb. & Crous and *P*. *leptorrhynchum* (Durieu & Mont.) D. Gramaje, L. Mostert & Crous. For *P*. *vibratile* (Fr.) D. Gramaje, L. Mostert & Crous only ITS2 is available, which contains several sequencing errors and was therefore not included. We used ITS sequences of the two strains CBS 110118 and CBS 110368 of *P*. *krajdenii*, which were confirmed by Mostert et al. [6] to be conspecific with the ex-type strain CBS 109479 based on actin and β-tubulin sequence data.

### Nomenclature

The electronic version of this article in Portable Document Format (PDF) in a work with an ISSN or ISBN will represent a published work according to the International Code of Nomenclature for algae, fungi, and plants, and hence the new names contained in the electronic publication of a PLOS article are effectively published under that Code from the electronic edition alone, so there is no longer any need to provide printed copies.

In addition, new names contained in this work have been submitted to MycoBank from where they will be made available to the Global Names Index. The unique MycoBank number can be resolved and the associated information viewed through any standard web browser by appending the MycoBank number contained in this publication to the prefix http://www.mycobank.org/MB/. The online version of this work is archived and available from the following digital repositories: [PubMed Central, LOCKSS].

## Results

### Phylogenetic results

**Fig 1:**
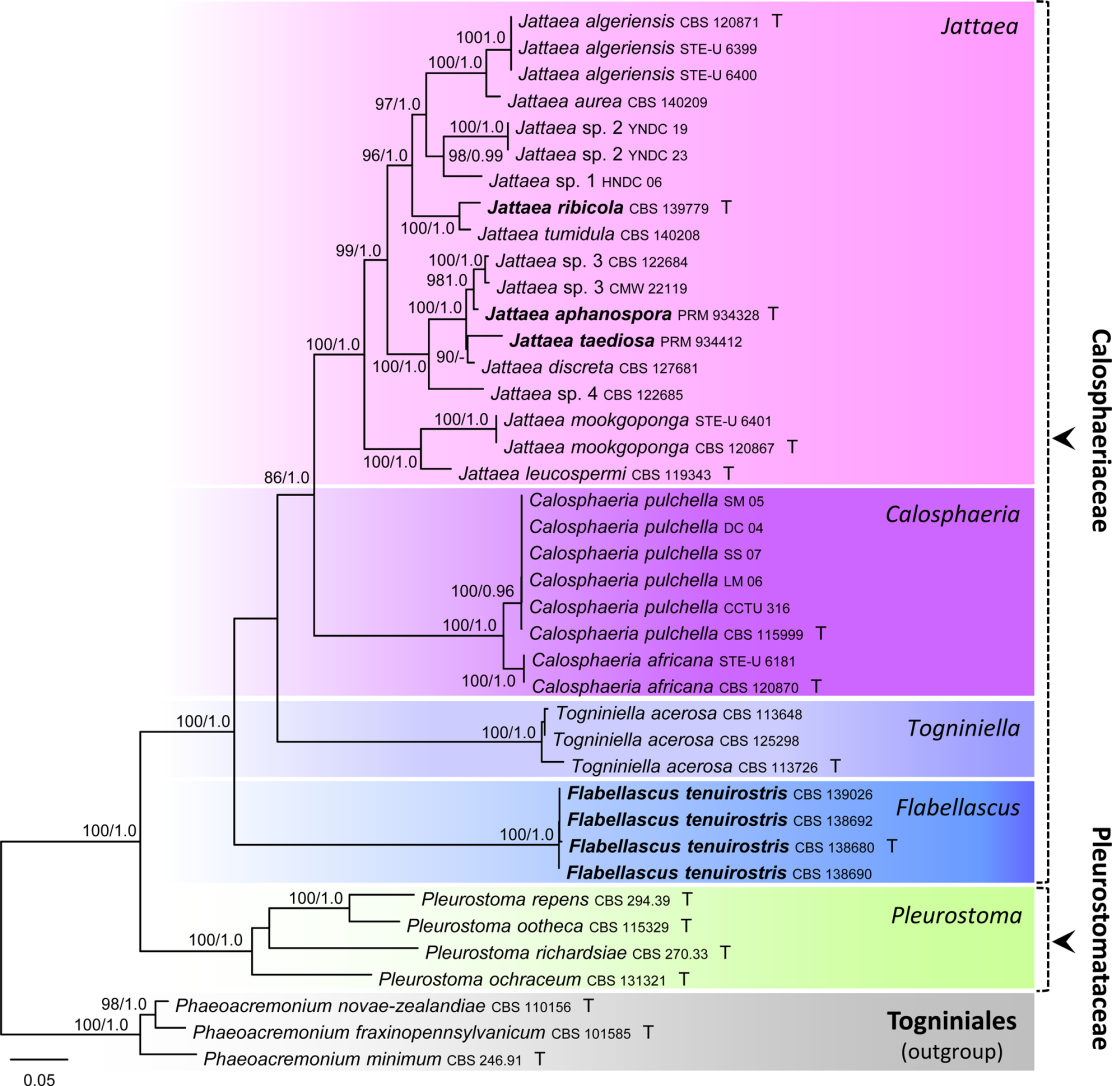
Phylogenetic analysis of the ITS-nuc18S-nuc28S-*rpb2*-β-tubulin sequences of the Calosphaeriales. Phylogram inferred from the ML analysis with RAxML using a GTRCAT model of evolution. Only high branch support is shown at the nodes, maximum likelihood bootstrap support (ML BS) ≥ 70% and Bayesian posterior probability (PP) ≥ 0.95. Taxa given in **bold** represent taxonomic novelties.

The first data sets consisted of 37 combined ITS, nuc18S, nuc28S, β-tubulin and *rpb2* sequences of members of the Calosphaeriales, each with 5 748 characters after introduction of gaps. The alignment had 1 587 distinct alignment patterns (ML analysis conducted with RAxML). In the ML tree shown in Fig 1, the Calosphaeriales was resolved as a robust monophyletic clade (100% ML BS / 1.0 PP) comprising five lineages that correspond to five strongly supported monophyletic genera in two families, the Calosphaeriaceae (100/1.0) and Pleurostomataceae (100/1.0). Four strains of the undescribed *Togniniella*-like fungus formed a monophyletic clade (100/1.0) positioned on a separate branch basal to the rest of taxa of the Calosphaeriaceae. Two other unknown species preliminarily assigned to *Jattaea* were positioned in this genus and are described as *J*. *aphanospora* and *J*. *ribicola* below. *Calosphaeria taediosa* grouped among species of *Jattaea* closely related to *J*. *discreta* (Berl.) Réblová.

**Fig 2:**
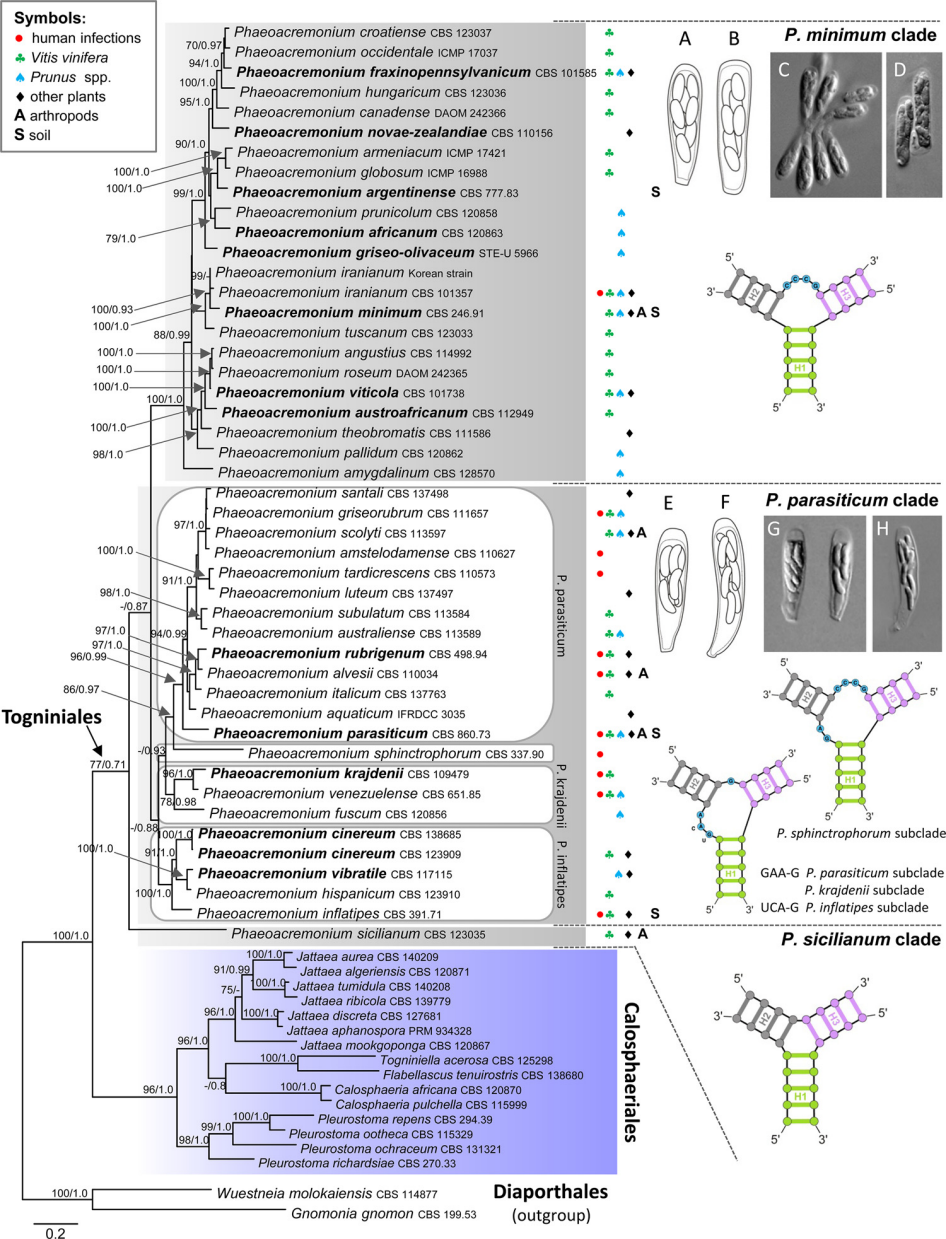
Phylogenetic analysis of the ITS-β-tubulin-actin sequences of 44 species of *Phaeoacremonium*. Phylogram inferred from the ML analysis with RAxML using a GTRCAT model of evolution. Only high branch support is shown at the nodes (MP BS ≥ 70%, PP ≥ 0.95). 2D diagram of a three-way junction composed of three helices labelled and colour-coded by H1 (green), H2 (grey), and H3 (purple) and the corresponding single stranded loop regions labelled L1 to L3 with nucleotides colour-coded in blue. The habitat of individual species is given according to Gramaje et al. [29] and illustrated with symbols and letters: ● human infections, ♣ *Vitis vinifera*, ♠ *Prunus* spp., ♦ other plants, A arthropods and S soil. Two subclades, *P*. *minimum* and *P*. *parasiticum*, are illustrated by asci and ascospores of four species: (A, C) *P*. *novae-zealandiae* (DAOM 35410). (B, D) *P*. *fraxinopennsylvanicum* (CBS 128920). (E, G) *P*. *cinereum* (PRM 934331). (F, H) *P*. *vibratile* (J.F. 04237). Taxa given in **bold** are known also as sexual morphs.

In the second analysis, the combined three gene data set of 63 ITS, actin and β-tubulin sequences consisted of 1 848 characters, belonging to 44 species of *Phaeoacremonium* and 15 species of the Calosphaeriales. The alignment had 1 210 distinct alignment patterns. In the ML tree shown in Fig 2, Togniniales is resolved as a well-supported clade (77/0.71). Three major clades containing *Phaeoacremonium* species were recovered and are labelled as *P*. *minimum*, *P*. *parasiticum* and *P*. *sicilianum* clades on the phylogram. The strongly supported *P*. *minimum* clade (100/1.0) encompasses 22 species. The *P*. *parasiticum* clade is statistically weakly supported (60/88), divided into four subclades labelled as *P*. *parasiticum* (96/0.99) with 13 species, *P*. *inflatipes* (100/1.0) with four species, *P*. *krajdenii* (78/0.98) containing three species and *P*. *sphinctrophorum* as a single species. *Phaeoacremonium sicilianum* is shown on a separate branch basal to all *Phaeoacremonium* species.

In the third ML analysis performed under the same options as the second one (results not shown), but without members of the Calosphaeriales, the inferred phylogram of the Togniniales contained the *P*. *minimum* clade (94) with *P*. *sicilianum* basal to it (92). The *P*. *parasiticum* clade was not resolved and instead five individual subclades were shown positioned separately on the tree, i.e. *P*. *inflatipes* (97), *P*. *parasiticum* (91) with *P*. *sphinctrophorum* basal to it (63), *P*. *krajdenii* (84) and *P*. *fuscum* on a separate branch.

### Consensus RNA secondary structure of ITS1

**Fig 3:**
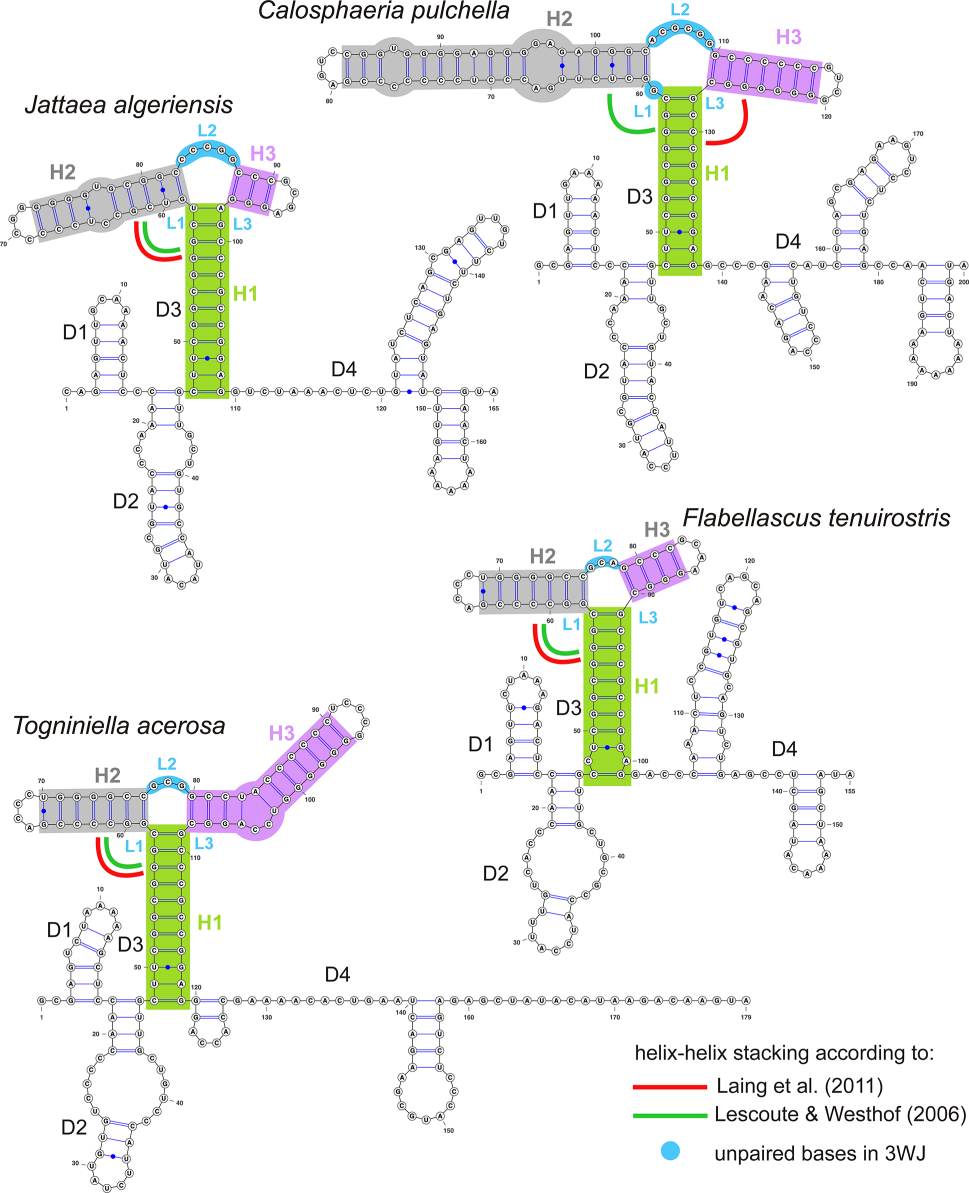
Predicted secondary structure models of ITS1 rRNA of type and representative species of genera of the Calosphaeriaceae. The predicted ‘ring’ models are transformed into linear models with helices separated into four domains labelled D1 to D4. A three-way junction in D3 composed of three helices labelled and colour-coded by H1 (green), H2 (grey), and H3 (purple) and the corresponding single stranded loop regions labelled L1 to L3 with nucleotides colour-coded in blue.

**Fig 4:**
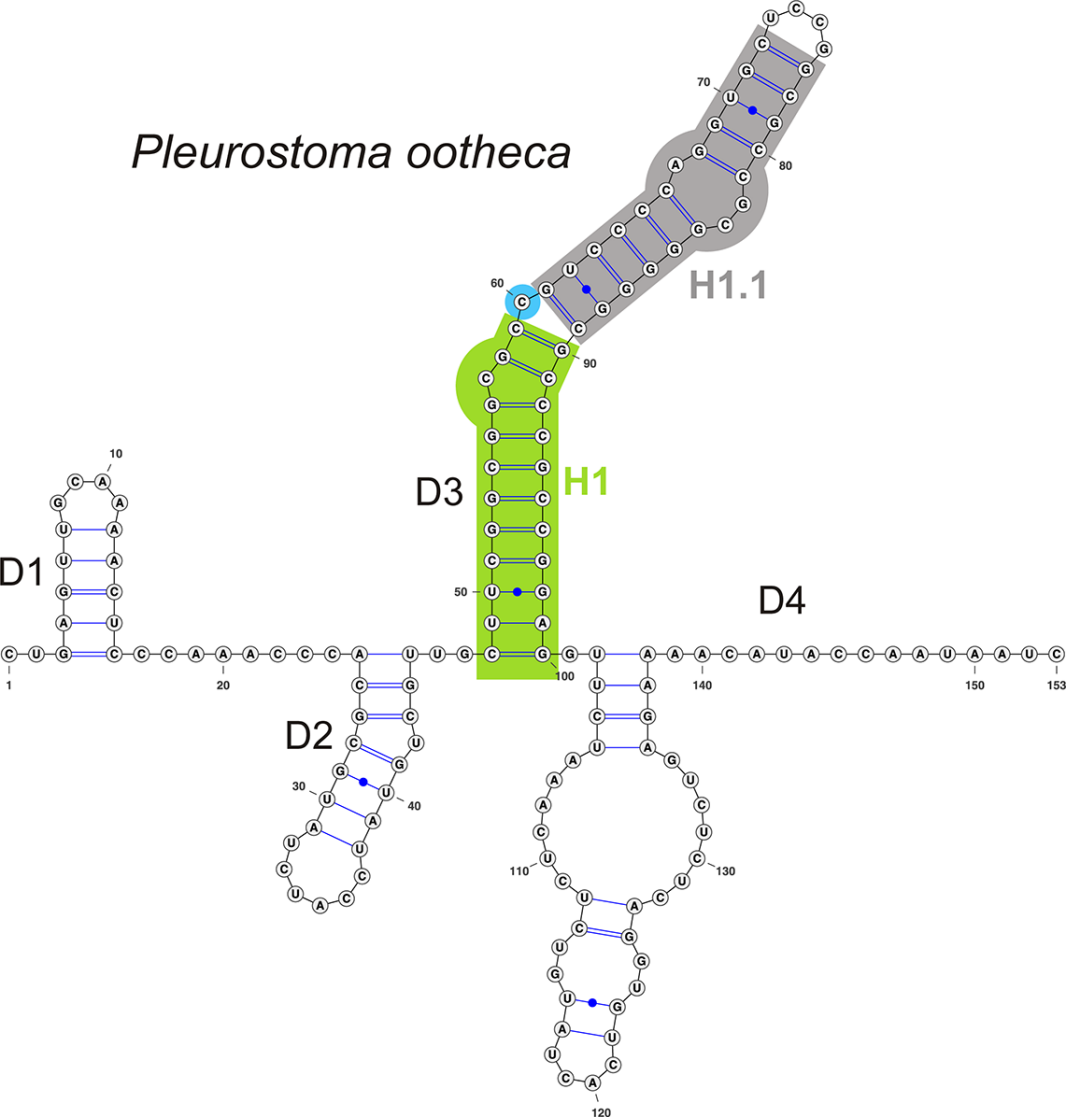
Predicted secondary structure model of ITS1 rRNA of *Pleurostoma ootheca* of the Pleurostomataceae. Symbols and colours as in Fig 3.

**Fig 5:**
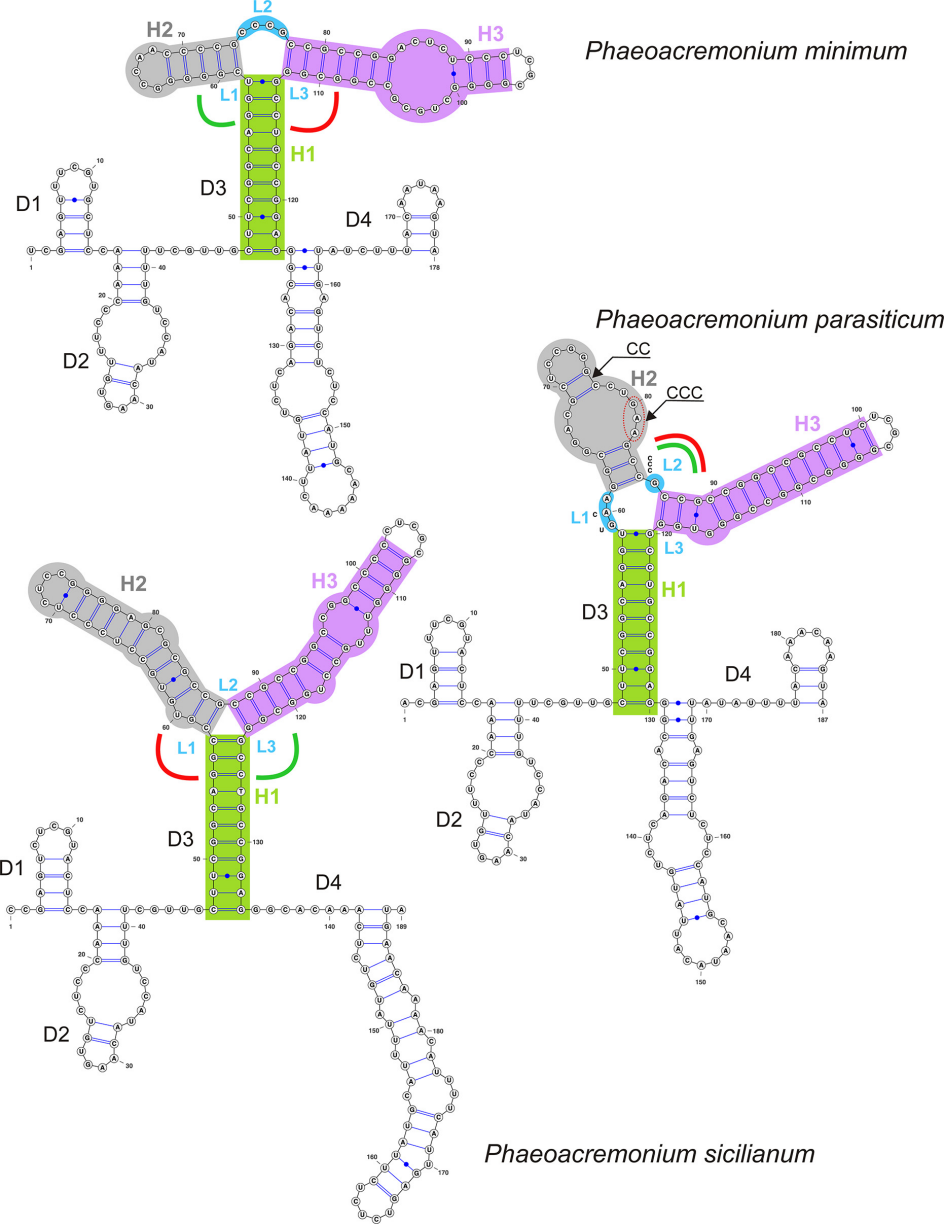
Predicted secondary structure models of ITS1 rRNA of *Phaeoacremonium minimum*, *P*. *parasiticum* and *P*. *sicilianum*. Symbols and colours as in Fig 3.

In all members of the Calosphaeriales and Togniniales the consensus 2D structure of ITS1 is folded into a ring structure with four domains (D1–D4) modelled for type or representative species of five calosphaeriaceous genera (Figs 3 and 4) and for representatives of the three major clades distinguished in the Togniniaceae, *i*.*e*. *P*. *minimum*, *P*. *parasiticum* and *P*. *sicilianum* (Fig 5). The number of nucleotides in each domain and discovered patterns of unpaired nucleotides in three-way junction (3WJ) are listed in Table 1.

**Table 1:** Number of nucleotides in D1–D4 domains of ITS1 rDNA of members of the Calosphaeriales and Togniniales.

Taxa	total nt	D1	D2				D3				D4	3WJ family	
				H1	L1	H2	L2	H3	L3	H1.1		[54]	[55]
***Calosphaeria***	**196–197**	**16**	**31**	**22**		**41–44**		**18**		**–**	**59–62**	C/H1-H3	A/H1-H2
* Calosphaeria africana*	196	16	31	22	**G**	41	**ACGCG**	18	**–**	**–**	62		
* Calosphaeria pulchella*	197	16	31	22	**G**	44	**ACGCGG**	18	**–**	**–**	59		
***Jattaea***	**164–173**	**16**	**30–31**	**24**	**–**	**23–31**		**10**		**–**	**53–61**	C/H1-H2	A/H1-H2
* Jattaea algeriensis*	165	16	30	24	**–**	24	**CCCGG**	10	**–**	**–**	56		
* Jattaea aphanospora*	164	16	31	24	**–**	27*	**CCTGG**	10	**–**	**–**	53		
* Jattaea aurea*	163	16	30	24	**–**	23	**CCCGG**	10	**–**	**–**	55		
* Jattaea discreta*	166	16	31	24	**–**	25*	**CCTGG**	10	**–**	**–**	53		
* Jattaea leucospermi*	175	16	30	24	**–**	31*	**CCTGG**	10	**–**	**–**	61		
* Jattaea mookgoponga*	171	16	31	24	**–**	25*	**CCTGG**	10	**A**	**–**	61		
* Jattaea ribicola*	167	16	30	24	**–**	27	**CCTGG**	10	**–**	**–**	55		
* Jattaea taediosa*	166	16	31	24	**–**	25*	**CCCGG**	10	**–**	**–**	55		
* Jattaea tumidula*	168	16	30	24	**–**	28	**CCTGG**	10	**–**	**–**	55		
* Jattaea* sp. 1	165	16	30	24	**–**	24	**CCCGG**	10	**–**	**–**	56		
* Jattaea* sp. 2	169	16	30	24	**–**	26	**CCAGG**	10	**–**	**–**	58		
* Jattaea* sp. 3	165	16	31	24	**–**	25*	**CCTGG**	10	**–**	**–**	54		
* Jattaea* sp. 4	164	16	31	24	**–**	23*	**CCTGG**	10	**–**	**–**	55		
***Togniniella acerosa***	**179**	**17**	**30**	**22**	**–**	**18**	**GCG**	**29**	**–**	**–**	**60**	C/H1-H2	A/H1-H2
***Flabellascus tenuirostris***	**156–157**	**17–18**	**29**	**22**	**–**	**18**	**GCA**	**12**		**–**	**54**	C/H1-H2	A/H1-H2
***Pleurostoma***	**154–176**	**15–16**	**30–32**	**24–25**		**–**	**–**	**–**	**–**	**29–47**	**53–57**	**–**	**–**
* Pleurostoma ootheca*	154	16	31	24	**C**	–	**–**	–	**–**	29	53		
* Pleurostoma ochraceum*	176	16	30	25	**C**	–	**–**	–	**–**	47	57		
* Pleurostoma repens*	165	16	32	24	**C**	–	**–**	–	**–**	32	55		
* Pleurostoma richardsiae*	163	15	31	24	**C**	–	**–**	–	**–**	38	54		
***Phaeoacremonium minimum*** clade	**168–186**	**15**	**31–32**	**22**	**–**	**16–18**	**CCCG**	**24–40**	**–**	–	**50–56**	C/H1-H3	A/H1-H2
***Phaeoacremonium parasiticum*** clade	**185–192**	**15**	**32**	**22**		**24–28**		**33–37**	**–**	–	**54–57**	C/H2-H3	A/H2-H3
* P*. *inflatipes* subclade	186–189	15	32	22	**TCA**	25	**G**	33–36	**–**	–	54–55		
* P*. *krajdenii* subclade	185–192	15	32	22	**GAA**	28	**G**	29–35	**–**	–	55–57		
* P*. *parasiticum* subclade	185–191	15	32	22	**GAA**	24–26	**G**	33–37	**–**	–	55–57		
* P*. *sphinctrophorum* subclade	190	15	32	22	**GA**	29	**CCCG**	30	**–**	–	56		
***Phaeoacremonium sicilianum*** clade	**189**	**15**	**31**	**22**	**–**	**30**	**–**	**36**	**–**	–	**55**	C/H1-H2	B/H1-H3

D1–D4 = domains in ITS1; H1–H4, H1.1 = helices in three-way junction; L1–L3 = unpaired nucleotides in three-way junction loop; nt = nucleotides; H2 * = an asterisk in nine *Jattaea* spp. indicates substitution in the first bp of helix H2 that leads to the formation of a non-canonical pair (G = C to G/A); [54 and 55] = classification of the topology of three-way junction using available programs Junction Explorer [54] and Cartaj [55].

The number of canonical and non-canonical base pairs and topology of D1 and D2 remain more or less identical among all analysed taxa. D1 consisted of a short helix and hairpin loop, D2 consisted of a helix with hairpin loop and internal symmetrical loop in members of the Calosphaeriaceae and Togniniaceae, while in all members of the Pleurostomataceae the internal loop is lacking.

D3 is the longest and highly variable domain, which exhibits two different topologies. In the Calosphaeriaceae, D3 forms a three-way junction; the three arms are labelled as helices H1 to H3 with unpaired nucleotides L1 to L3 on the junction loop (Fig 3). In the Pleurostomataceae, the 3WJ is lacking and instead a long duplex labelled H1.1 is formed as a continuation of H1 (Fig 4). The topology of D3 and the number of unpaired nucleotides on the junction loop are genus-specific in the Calosphaeriales.

In eleven strains of *Jattaea* representing nine species we detected two types of substitutions in the first two bp of H2 that lead to the formation of a non-canonical pair in seven of them. These substitutions correlate with recovered subclades of *Jattaea* (Fig 1). The canonical and ‘wobble’ pairs G = C, U/G in *J*. *algeriensis*, *J*. *aurea* and *Jattaea* sp. 1 and *Jattaea* sp. 2 change to wobble and canonical pairs G/U, C = G in *J*. *tumidula* and *J*. *ribicola*, while in the remaining species the substitution leads to G/A, C = G pairs. The formation of the first non-canonical G/A pair causes shortening of the H2 helix and the unpaired nucleotides become part of the L1 and L2. For details see Table 1.

In the Togniniaceae we discovered a pattern identical to that observed in the Calosphaeriaceae with the exception that domains D2 and D3 are always separated by a single-stranded region (Fig 5). The 3WJ in D3 occurs in all *Phaeoacremonium* species. Its overall topology, position of unpaired nucleotides on the junction loop or their complete absence, and the length of H2 and H3 characterise species in each of the three major clades. Moreover, in the *P*. *parasiticum* clade we discovered three alternative models of D3 that characterise four recovered subclades (Figs 2 and 5). The helix H2 is the most variable of the three helices in 3WJ. It is conserved in all members of the *P*. *minimum* clade, but highly variable among subclades of the *P*. *parasiticum* clade. The main changes comprise CC insertion in the 3’-half of H2 in all three species of the *P*. *krajdenii* subclade and a substitution in the 3’-half of the internal loop in H2 such as GAA for CCC in all members of the *P*. *parasiticum* subclade and GTG for CCC in *P*. *sphinctrophorum* (Fig 5). The 5’-half of H2 exhibits several substitutions and a deletion in the *P*. *inflatipes* and *P*. *sphinctrophorum* subclades. Moreover, the three major *Phaeoacremonium* clades are also characterised by the length of ITS1 sequences. For example species grouped in the *P*. *minimum* clade have ITS1 sequences generally shorter (168–186 nt) than species of the *P*. *parasiticum* clade (185–192 nt). The length of the ITS1 sequence of *P*. *sicilianum* is 189 nt.

When modelling the 3WJ for *P*. *sicilianum* Essakhi et al., we discovered a possible mismatch in the second bp of H1 that may indicate a sequencing error. The 2D structure is more conserved than the primary structure. Therefore, we assume that the substitution of A for G only in *P*. *sicilianum*, i.e. C = G changed to a non-canonical C/A pair, disrupts the first pair in H1, which is otherwise a highly conserved structure in all *Phaeoacremonium* species and also in all members of the Calosphaeriales.

The D4 domain is genus-specific in members of the Calosphaeriales; it contains 2–3 duplexes with variously positioned bulges and internal loops and hairpin loops of different length. Similarly, three phylogenetic groups distinguished in *Phaeoacremonium* are characterised by a distinct topology of the D4 domain. Species of the *P*. *minimum* and *P*. *parasiticum* clades possess two duplexes, while a long single duplex at the 3’-end characterises the *P*. *sicilianum* clade.

### Consensus RNA secondary structure of ITS2

**Fig 6:**
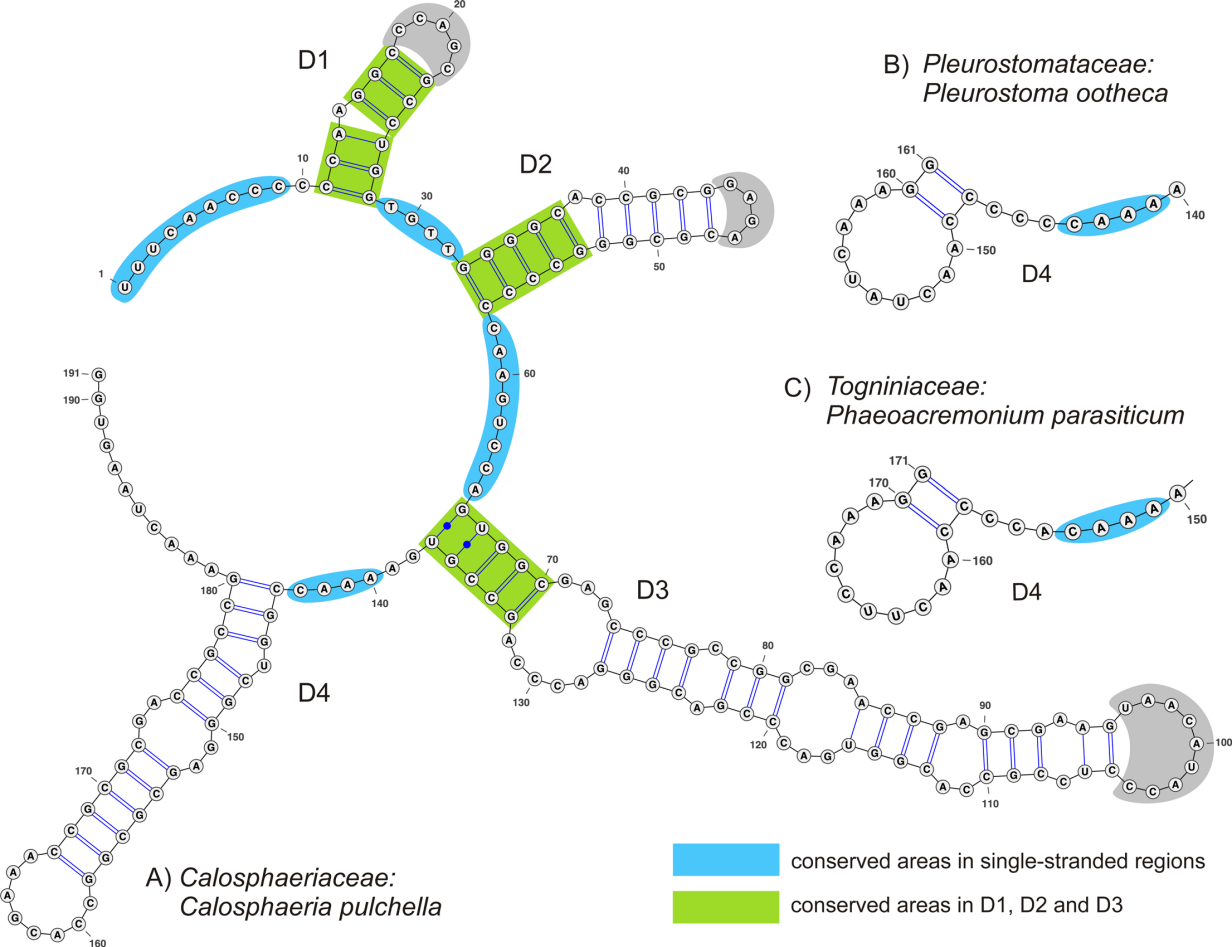
Predicted secondary structure model of ITS2 rRNA of *Calosphaeria pulchella*. The ‘ring’ model is composed of four domains labelled D1 to D4. The basal parts of D1–D3 colour-coded by green and single-stranded areas colour-coded by blue represent conserved areas in all members of the Calosphaeriales and Togniniales. D4 domain of the: (A) Calosphaeriaceae. (B) Pleurostomataceae. (C) Togniniaceae.

The consensus secondary structure of the ITS2 is folded into a ring structure with four domains (D1–D4) separated by single-stranded regions. The sequence of *Calosphaeria pulchella* was mapped on the 2D structure model of ITS2 with highlighted conserved areas occurring in the single-stranded regions and in the basal parts of duplexes in D1, D2 and D3 domains in all members of the Calosphaeriales and Togniniales (Fig 6).

D1 and D2 consist of a conserved stem and a hairpin loop with a variable number of nucleotides. D3 is the longest duplex in the ITS2. It consists of four to five internal loops, occasionally a bulge at the 3’-end (*Togniniella*, *Phaeoacremonium*) and a hairpin loop of variable length.

The fourth duplex D4 is highly variable. Based on its topology it is similar among members of the Calosphaeriaceae on one hand and Pleurostomataceae and Togniniaceae on the other, exemplified by the presence of internal loops, bulges and number of canonical C = G and A-U pairs vs. wobble G/U pairs in the stem. In *Calosphaeria*, *Jattaea* and *Togniniella* the duplex in D4 is positioned in the middle and contains one internal loop, whilst the internal loop is lacking in *Flabellascus*. *Pleurostoma* and *Phaeoacremonium* possess almost identical D4; the short helix is always positioned at the 3’-end.

The alternative duplex at the 3’-end of the D4 domain, which can occur besides the regular duplex, was predicted only for *Flabellascus*. It is equivalent to the duplex of members of the Pleurostomataceae and Togniniaceae due to its position on a ring.

### Taxonomy

Multilocus phylogenetic analysis of members of the Calosphaeriales revealed five distinct strongly supported lineages at the genus level, namely *Calosphaeria*, *Jattaea*, *Pleurostoma* and *Togniniella*. Four strains of the undescribed *Togniniella*-like fungus forming a monophyletic clade are introduced as a new genus *Flabellascus* below. Two new species of *Jattaea*, *J*. *aphanospora* and *J*. *ribicola*, are described and *C*. *taediosa* is combined in *Jattaea*. The asexual morphs of *J*. *aurea* and *J*. *tumidula* and the sexual morph of *Phaeoacremonium cinereum* of the Togniniales are reported for the first time.

***Flabellascus*.** Réblová, gen. nov.

[urn:lsid:indexfungorum.org:names:814416]

*Ascomata* non-stromatic, immersed, only necks emerging, venter subglobose with a long neck. Ostiole periphysate. *Ascomatal wall* leathery to fragile, two-layered. *Paraphyses* persistent, septate, inflated between the septa, tapering upwards, hyaline, longer than the asci. *Ascogenous hyphae* elongated, branched, with ellipsoidal to obpyriform minute cells. *Asci* unitunicate, 8-spored, clavate, stipitate, conspicuously thickened at the apex, lacking a distinct discharge mechanism, arranged in a spicate formation on ascogenous hyphae. Ascospores suballantoid, hyaline, smooth, non-septate, arranged in a fascicle in the upper part of the ascus. *Conidiophores* macronematous to semi-macronematous, dark brown, unbranched, ending in a terminal phialide or 2–3 verticillate phialides, or giving rise to a series of short, dark brown branches which terminate in a single or 2–3 phialides. *Phialides* ampulliform or elongate-ampulliform, hyaline to pale translucent brown, gradually tapering, with a flaring collarette. *Conidia* hyaline, non-septate, of two types; type I allantoid, type II suballantoid to reniform.

Etymology. *Flabellum* (L) fan, referring to the fan-shape arrangement of asci on ascogenous hyphae.

Type species. *Flabellascus tenuirostris* Réblová

Comments. *Flabellascus* bears a strong resemblance to *Togniniella* in morphology of ascomata, asci and ascospores. It can be distinguished from the latter taxon by morphological characters of conidiophores, conidiogenous cells and two types of conidia formed *in vitro*.

**Fig 7:**
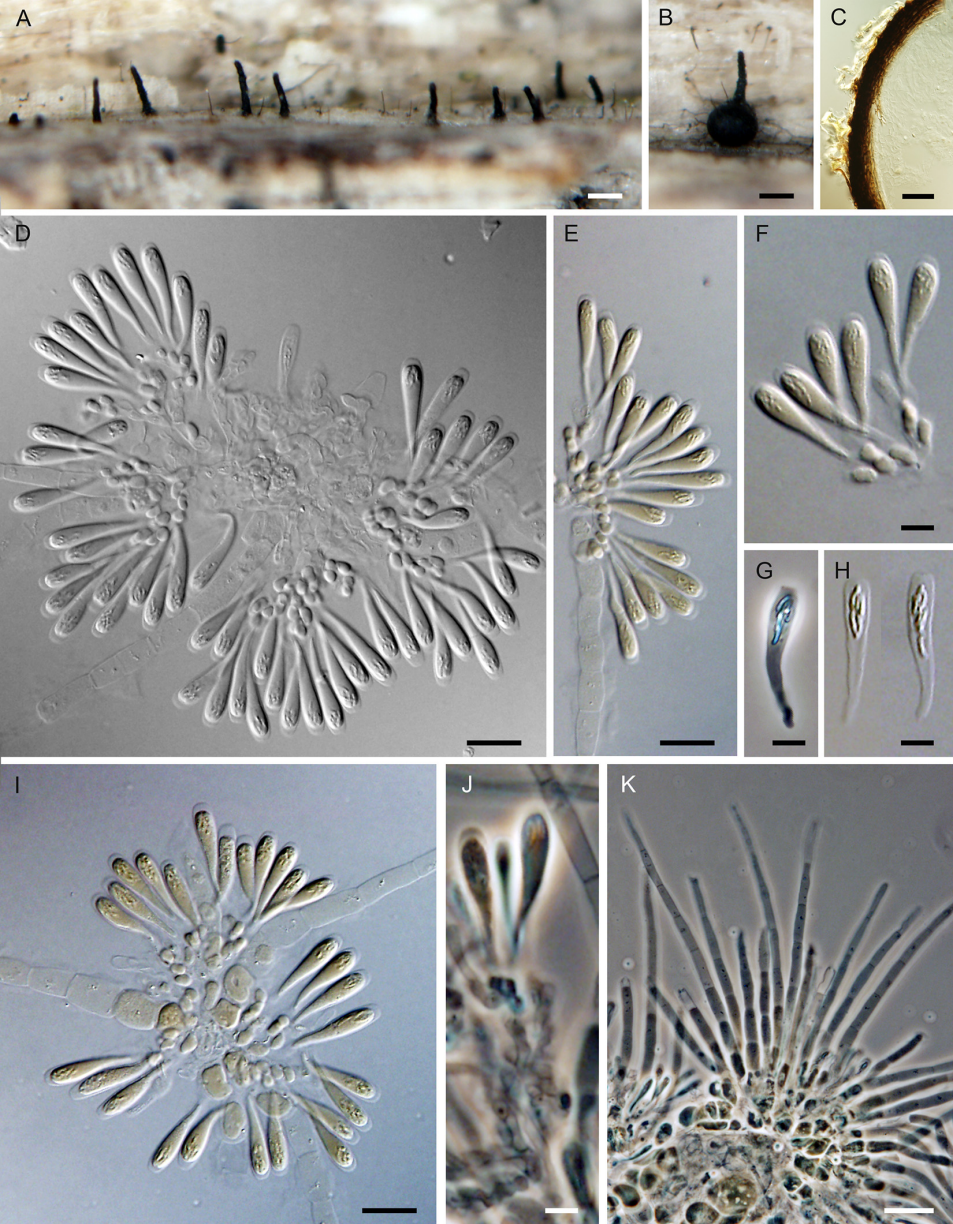
*Flabellascus tenuirostris*, a lignicolous species with long projecting necks. (A, B) Ascomata. (C) Vertical section (lateral part) of the ascoma. (D–F, I) Asci attached to ascogenous hyphae in spicate or fan-like arrangements. (G, H) Asci. (J) Detail of ascogenous hypha. (K) Paraphyses. DIC (C–F, H, I), PC (G, J, K), bar = 250 μm (A, B), 25 μm (C), 10 μm (D, E, I), 5 μm (F–H, J), 20 μm (K). PRM 934327 holotype (A, B, F), M.R. 3691 (C, G, H, J, K), M.R. 3815 (D, E), M.R. 3821(I).

**Fig 8:**
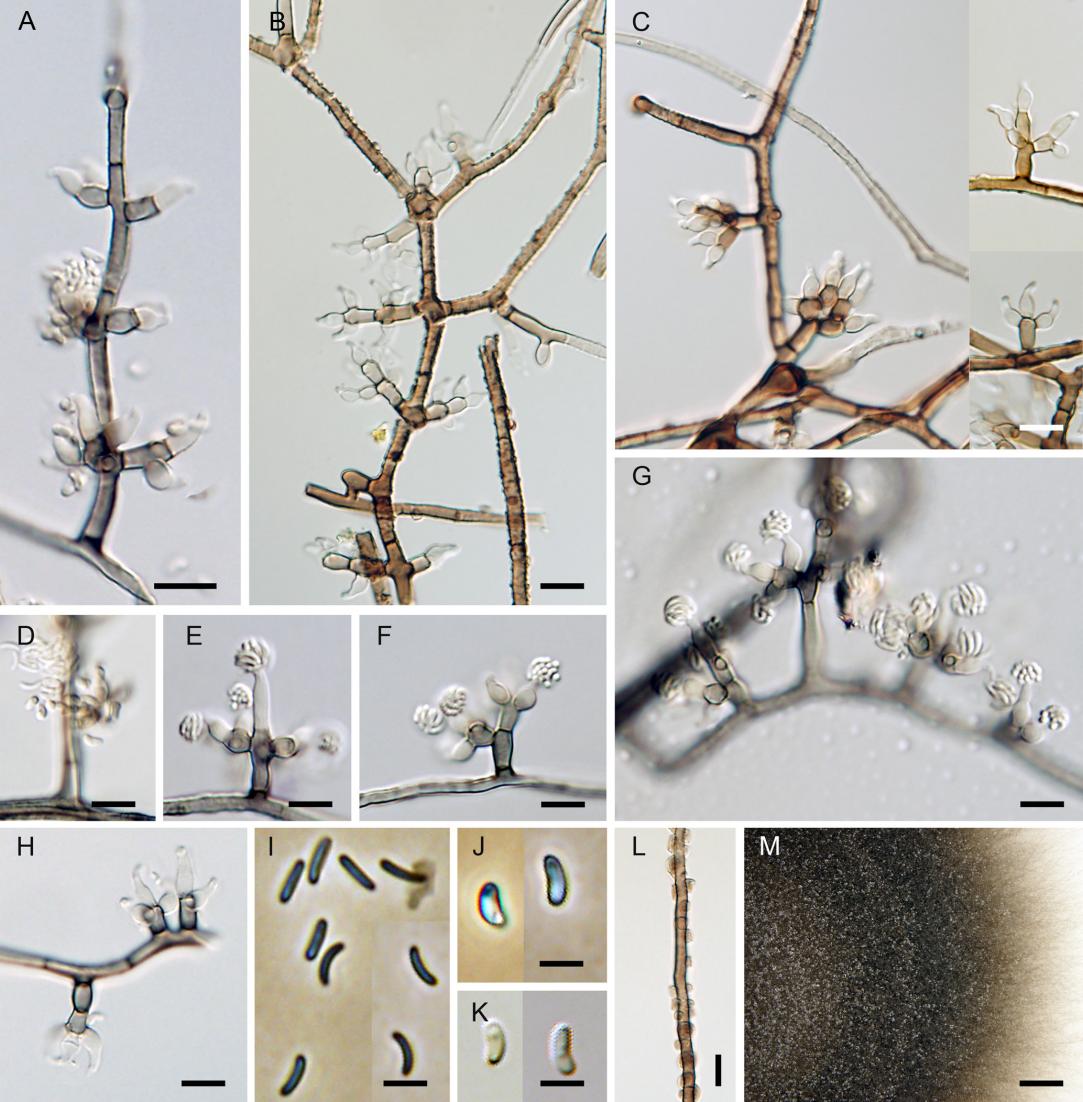
*Flabellascus tenuirostris* with conidiophores and conidia of two types. (A–C, H) Conidiophores with phialides in whorls. (D) Phialides with type I and type II conidia. (E–G) Phialides with type I conidia aggregated in slimy heads. (I) Type I conidia. (J, K) Type II conidia. (L) Aerial hypha with prominent exudate droplets observed as warts. (M) Colony. DIC (A–H, K, L), PC (I, J), bar = 10 μm (A–H, L), 5 μm (I–K), 0.5 cm (M). CBS 138690 (A, M on MLA 14 d), CBS 138680 (B, C, K on PCA 14 d), CBS 139026 (D–H, J, K on PCA, 14 d), CBS 138692 (L on MLA 14 d).

***Flabellascus tenuirostris*.** Réblová, comb. nov. (Figs 7A–7K, 8A–8O)

[urn:lsid:indexfungorum.org:names:814417]

*Ascomata* non-stromatic, immersed in decaying wood, only necks emerging, scattered to gregarious; venter 250–370(–400) μm diam, 230–380 μm high, subglobose, dark brown, at the bottom with sparse dark brown, septate, branched, 2.5–3.5 μm wide hyphae; neck central, 60−80 μm wide, 200–400 μm long, cylindrical, straight or slightly flexuous, rounded at the apex, with sparse, subhyaline to pale brown, non-septate, unbranched, 2.5–3.0 μm wide and up to 15 μm long hairs radiating from the surface. *Ostiole* periphysate. *Ascomatal wall* 20–30 μm thick, leathery to fragile, two-layered. Outer layer consisting of brown *textura prismatica* to *t*. *angularis*, towards the interior grading into several layers of thin-walled subhyaline to hyaline flattened cells. *Paraphyses* persistent, septate, inflated between the septa, hyaline, ca. 4.0–6.0 μm wide near the base, ca. 2.0–2.5 μm wide at the apex, longer than the asci. *Ascogenous hyphae* elongated, branched, with ellipsoidal to obpyriform cells 2.5–3.0 μm wide, 4.0–5.5 μm long, growing in sympodial succession. *Asci* (18–)19–24.5(–26) × 4.0–5.5 μm (mean ± SD = 23.8 ± 1.8 × 4.5 ± 0.4 μm), with sporiferous part 6.0–9.5 μm long (mean ± SD = 8.4 ± 1.1 μm), L/W 5:1, clavate, tapering towards the base, in spicate or fan-shaped clusters on ascogenous hyphae; apex broadly rounded to obtuse, thickened to 1.0–1.5 μm, lacking a distinct discharge mechanism. Asci floating freely in the centrum upon maturation, 8-spored. *Ascospores* (3.0–)3.5–4.5 × 0.7–1.0 μm (mean ± SD = 3.8 ± 0.4 × 0.9 ± 0.2 μm), suballantoid with rounded ends, hyaline, smooth, non-septate, arranged in a fascicle in the upper part of the ascus.

Characters in culture. Colonies on MLA reaching a radius of 30–35 mm after 14 d at 25°C, circular, flat, felty, dense, with filiform margins. Aerial mycelium abundant, colony surface brown-grey (oac723) in the centre, brown (oac768) towards the margin; reverse dark brown (oac768). Mycelium consisting of branched, septate, medium to dark brown hyphae, 3.5–4.5 μm wide, smooth-walled, frequently with prominent exudate droplets observed as warts. *Conidiophores* macronematous or semi-macronematous of variable length, 12–65(–89) μm long, 2.5–3.5 μm wide, arising from aerial hyphae, erect, straight or flexuous, one to several-septate, slightly constricted at the septa, dark brown, ending in a terminal phialide or in a whorl of 2–3 phialides, or giving rise to a series of short branches, which terminate in a single or several verticillate phialides. *Phialides* (5.0–)6.0–9.0(–11) × 2.5–3.5 μm (mean ± SD = 7.1 ± 1.4 × 3.1 ± 0.3 μm), ampulliform or elongate-ampulliform, gradually tapering to 1.0–1.5 μm, with a narrow, often slightly bent neck; collarette inconspicuous, flaring 1.5–3.0 μm wide. Phialides hyaline or translucent pale brown, their supporting cells dark to medium brown, subhyaline to hyaline towards the tip, terminal and lateral, up to three phialides inserted laterally between septa of the conidiophore. *Conidia* aggregating in slimy heads at the apex of the phialides, hyaline, non-septate, smooth, of two types: type I conidia narrowly allantoid, 3.0–3.5 μm long, about 1.0 μm wide (mean ± SD = 3.2 ± 0.3 × 0.9 ± 0.1 μm), type II conidia suballantoid to reniform, slightly tapering towards the basal end, 3.0–3.5 × 1.5–2.0 (mean ± SD = 3.1 ± 0.3 × 1.6 ± 0.3) μm.

Holotype. Czech Republic. Southern Bohemia: Novohradské hory Mts., Horní Stropnice, Hojná Voda National nature monument, decaying wood of *Fagus sylvatica*, 4 Oct 2012, M. Réblová M.R. 3763 (PRM 934327, holotype, ex-type culture CBS 138680).

Etymology. *Tenuis* (L) thin, *rostrum* (L) beak, referring to ascomata with a slender projecting ostiole.

Additional specimens examined. Czech Republic. Southern Bohemia: Novohradské hory Mts., Horní Stropnice, Hojná Voda National nature monument, decaying wood of *Fagus sylvatica*, 4 Oct 2012 *M*. *Réblová* M.R. 3764; ibid. 13 Oct. 2013, *M*. *Réblová* M.R. 3815 (culture CBS 138690), M.R. 3821 (culture CBS 138692); ibid. 14 Oct 2013, M. Réblová M.R. 3829 (culture CBS 139026). Czech Republic. Southern Moravia: Břeclav distr., Valtice, Rendezvous Valtice National nature monument, decaying wood of *Quercus cerris*, 17 Nov 2012, M. Réblová M.R. 3691 (culture CBS 137795).

Comments. Two types of conidia were observed *in vitro*. In the strain CBS 139026 (PCA, 30 d) mostly the narrower allantoid type I conidia were formed and remained attached in slimy droplets to the tips of phialides, while these and the wider, suballantoid to reniform type II conidia were observed rarely at the same time (Fig 8D–8G). It is probable that the type I conidia are formed first, followed by formation of slightly wider and shorter conidia of type II, which are abundantly present in older cultures. However, we cannot rule out the possibility that the type I conidia swell to produce type II conidia.

Vegetative hyphae were frequently seen with droplets of exudate observed as warts of various sizes (Fig 8L). The droplets often fuse especially in loops formed on vegetative mycelium. Similar droplets are often seen in species of *Phaeoacremonium* [6].

Examination of the holotype (NY 00911861) of *Ceratostomella microspora* Ellis & Everh. [56] showed that this species is morphologically very similar to *F*. *tenuirostris*, but in absence of fresh material and DNA sequence data a possible conspecificity cannot be proven (see Discussion).

**Fig 9:**
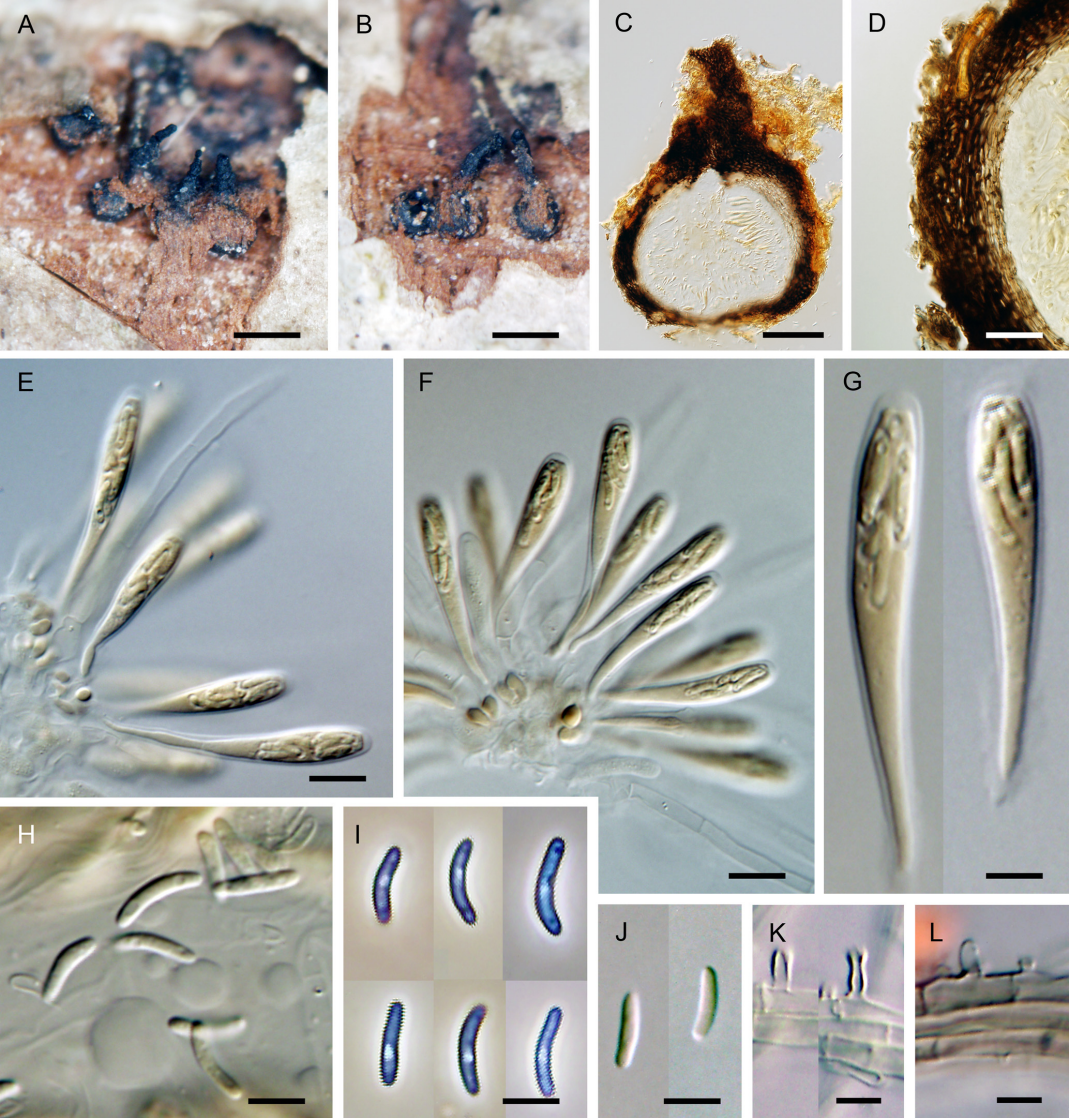
*Jattaea aphanospora*, a lignicolous species on the host and in culture. (A, B) Ascomata. (C, D) Vertical section (lateral parts) of the ascoma. (E–G) Asci. (H, I) Ascospores. (J) Conidia. (K, L) Adelophialides. DIC (C–H, J–L), PC (I), bar = 500 μm (A, B), 100 μm (C), 20 μm (D), 5 μm (E–L). PRM 934328 holotype (A–L, J–L on MLA 14 d).

***Jattaea aphanospora*.** Réblová & J. Fourn., sp. nov. (Fig 9A–9L)

[urn:lsid:indexfungorum.org:names:814418]

*Ascomata* non-stromatic, growing solitarily or in small loose valsoid groups of 3–6 individuals on wood beneath the periderm; venter 250–350 μm diam, 250–380 μm high, laterally collapsing, globose to subglobose, dark brown, glabrous; neck central, cylindrical, 80–90 μm wide, 250–500 μm high, rounded at the apex, dark brown, glabrous. *Ostiole* periphysate. *Ascomatal wall* 25–35 μm thick, fragile, two-layered. Outer layer consisting of brown *textura prismatica* to *t*. *epidermoidea*, towards the interior grading into several layers of thin-walled subhyaline to hyaline, flattened cells. *Paraphyses* persistent, septate, non-constricted at the septa, 2.5–3.5 μm wide, ca. 2.0–2.5 μm wide at the apex, longer than the asci. *Ascogenous hyphae* discrete, branched, with obovoid to obpyriform, 2.5–3.5 μm wide, 3.5–5.0 μm long cells formed in sympodial succession. *Asci* 30–38(–42) × (5.0–)5.5–6.0 μm (mean ± SD = 35.5 ± 4.5 × 5.7 ± 0.4 μm), with sporiferous part (14–)15.5–20(–23) μm long (mean ± SD = 17.6 ± 2.7 μm), clavate, stipitate, gradually tapering toward the base; apex obtuse to broadly rounded, thickened to 1.0–1.5 μm, lacking a distinct discharge mechanism. Asci floating freely in the centrum upon maturation, with a bristle-like appendage at the base, 8-spored. *Ascospores* (6.0–)6.5–7.5(–8.0) × 1.5(–2.0) μm (mean ± SD = 7.1 ± 0.6 × 1.5 ± 0.2 μm), suballantoid to oblong, slightly curved, hyaline, smooth, non-septate, 2–3-seriately arranged.

Characters in culture. Colonies on MLA reaching a radius of 15–20 mm after 14 d at 25 °C, circular, flat, felty, with entire margins. Aerial mycelium medium to sparse; colony surface white (oac909) to beige (oac809) near the centre, almost white towards the margin, reverse grey (oac865) in the centre, white-grey to inconspicuous towards the margin. Mycelium consisting of branched, septate, hyaline to subhyaline hyphae, 1.5–2.5 μm wide, smooth-walled. *Conidiophores* micronematous, reduced to single conidiogenous cells. *Adelophialides* 3.0–5.5(–8.0) × 1.5–2.0 μm (mean ± SD = 4.8 ± 1.7 × 1.8 ± 0.1 μm), hyaline, subcylindrical, sometimes slightly attenuated at the base, tapering to 1.0–1.5 μm below the collarette; collarette flaring, ca. 2.5–3.0 μm wide. *Conidia* 6.0–6.5(–7.3) × 1.5–2.0 μm (mean ± SD = 6.3 ± 0.2 × 1.8 ± 0.1) μm, oblong to suballantoid, slightly curved, hyaline, smooth, non-septate.

Holotype. France. Midi-Pyrénées: Ariège, Rimont, Las Muros, on decaying branch of *Crataegus* sp. still attached to the trunk, 9 May 2006, J. Fournier J. F. 06100 (PRM 934328, holotype).

Etymology. *Aphanos-* (Gk), inconspicuous or indistinct, referring to a difficulty to recognise this taxon among other *Jattaea* species based on ascospore characteristics.

Comments. Although this specimen was originally cited under *Jattaea discreta* [8], with increased taxon sampling it is positioned on a separate branch closely related to the two undescribed strains *Jattaea* sp. 3 from *Protea* spp. originating in South Africa [57]. *Jattaea aphanospora* differs from *J*. *discreta* by smaller asci and ascospores. The ascospores of *J*. *discreta* are also oblong to suballantoid and non-septate, but based on the revision of the isotype material (Saccardo Mycotheca Veneta no. 1450, NY), the ascospores measure 7.0–8.5 × 1.5–2.0 μm and asci 35–48(–50) × 6.0–7.0 μm, with sporiferous part 18–25(–28) μm long.

*Jattaea aphanospora* is comparable to *J*. *echinella* (Ellis & Everh.) Réblová [8], which differs by slightly shorter ascospores and shorter asci in the sporiferous part and by necks with a red-brown apex observed in a translucent light. The culture of *J*. *aphanospora* is no longer available due to poor growth of mycelium, which eventually stopped before the culture could be deposited.

**Fig 10:**
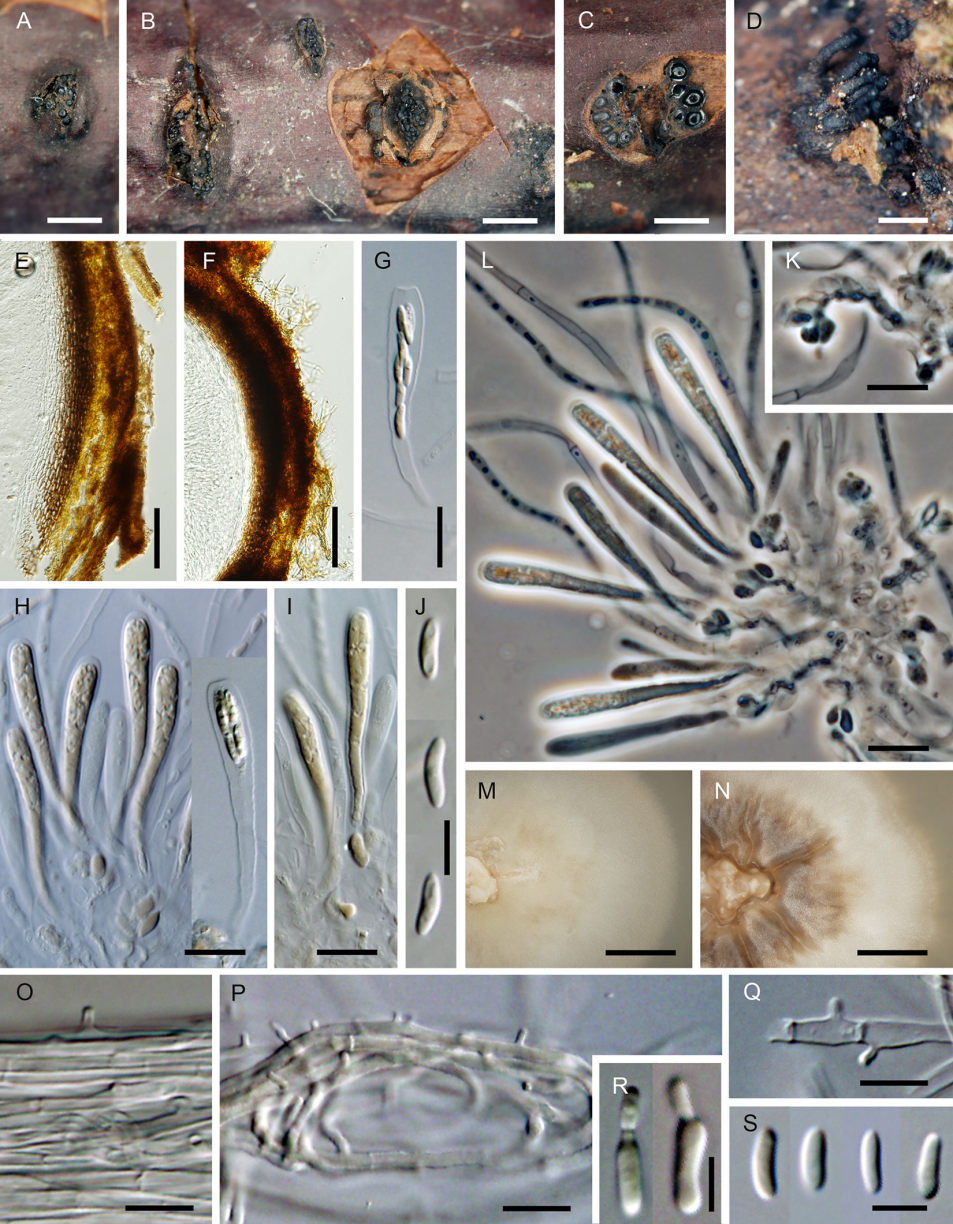
*Jattaea ribicola*, a lignicolous species with golden-brown tomentum. (A–D) Ascomata. (E, F) Vertical section of the ascoma. (G–I) Asci. (J) Ascospores. (K, L) Ascogenous cells, asci and paraphyses. (M, N) Colony. (O–Q) Adelophialides. (R) Inflated conidia producing additional conidia from a single phialidic opening. (S) Conidia. DIC (E–J, O–S), PC (K, L), bar = 1000 μm (A–C), 500 μm (D), 50 μm (E, F), 10 μm (G–I, K, L, O, P), 5 μm (J, R, S), 1 cm (M, N). PRM 934329 holotype (A–L), CBS 139779 ex-type (M, O–S on MLA 21 d, N on MLA 30 d).

***Jattaea ribicola*.** Réblová & Jaklitsch, sp. nov. (Fig 10A–10S)

[urn:lsid:indexfungorum.org:names:814419]

*Ascomata* typically densely aggregated in circinate groups of 8–16 individuals on wood beneath the periderm; venter 300–450 μm diam, 350–500 μm high, globose to subglobose, brown, covered by a dark yellow-brown or golden-brown tomentum, hyphae of the tomentum agglutinating beyond the ascomata as a compact, 30–45(–60) μm thick stromatic layer of 2.0–2.5 μm wide, densely intertwined, septate, unbranched or sparsely branched yellow-brown hyphae. Necks central, cylindrical, 100–120 μm wide, up to 750 μm long, dark brown to black, glabrous, straight or slightly flexuous, broadly rounded at the apex, upright or partly parallel to the substratum, then ascending, converging radially and collectively erumpent through a disc of dense tomentum, piercing the periderm in a narrow fissure. *Ostiole* periphysate. *Ascomatal wall* 30–45 μm thick, leathery to fragile, two-layered. Outer layer consisting of brown *textura angularis* to *t*. *prismatica*, towards the interior grading into several layers of thin-walled subhyaline to hyaline, flattened cells. *Paraphyses* persistent, septate, non-constricted at the septa, 2.0–3.5 μm wide, ca. 2.0 μm wide at the apex, longer than the asci. *Ascogenous hyphae* discrete, branched, with obovoid to obpyriform, 4.0–5.0(–5.5) μm long, 2.5–3.5 μm wide cells formed in sympodial succession. *Asci* (38–)41–46(–49) × 5.5–6.5(–7.0) μm (mean ± SD = 44.3 ± 2.7 × 6.1 ± 0.5 μm), with sporiferous part (16–)17.5–23(–24.5) μm long (mean ± SD = 20.3 ± 2.3 μm), clavate, stipitate, gradually tapering toward the base; apex obtuse to broadly rounded, thickened to 1.0–1.5 μm, lacking a distinct discharge mechanism. Asci floating freely in the centrum upon maturation, with a bristle-like appendage at the base, 8-spored. *Ascospores* (5.4–)6.0–7.0(–8.0) × 1.5–2.0(–2.2) μm (mean ± SD = 6.6 ± 0.6 × 1.9 ± 0.2 μm), ellipsoid or oblong, slightly curved, hyaline, smooth, non-septate, 2–3-seriately arranged.

Characters in culture. Colonies on MLA reaching a radius of 20–25 mm after 21 d at 25 °C, circular, flat, waxy or with a slightly moist appearance, with entire margins, developing several wrinkles and deep radial folds. Colony surface white (oac909) to ivory (oac816) to beige (oac781), becoming pale brown (oac770) to brown-red (oac768) in the centre, pale pink (oac795) towards the margin; reverse ivory (oac816), becoming brown-red (oac768) in the centre, white (oac909) towards the margin. Mycelium consisting of branched, septate, hyaline hyphae, subhyaline to translucent pale brown in mass, 1.5–2.5 μm wide, smooth-walled. Aerial hyphae often forming strands. *Conidiophores* micronematous, reduced to conidiogenous cells. *Adelophialides* 1.5–3.0 × 1.0–2.0 μm (mean ± SD = 1.8 ± 0.5 × 1.2 ± 0.3 μm), subcylindrical, tapering to 1.0–1.5 μm, lacking a visible collarette. *Conidia* 4.5–6.0(–7.5) × 1.5–2.0 μm (mean ± SD = 5.3 ± 0.8 × 1.7 ± 0.2) μm, hyaline, non-septate, oblong-ellipsoidal, slightly curved, smooth-walled. Mature conidia becoming inflated, forming new conidia from a single phialidic opening *in vitro*.

Holotype. Austria. Niederösterreich: Rax, near Seehütte, on decaying branches of *Ribes petraeum*, 21 Sep 2014, W. Jaklitsch (PRM 934329, holotype, ex-type culture CBS 139779).

Etymology. Ribicola, occurring on *Ribes* (L), referring to the host.

Additional specimen examined. Austria. Osttirol: Virgental, Prägraten, Umbalfälle, on branches of *Ribes petraeum*, 10 Sep 2001, W. Jaklitsch W.J. 1788 (WU 33578).

Comments. *Jattaea ribicola* forms relatively large circular groups of tightly aggregated ascomata beneath the periderm, a feature that is rather typical of members of *Calosphaeria*. Necks of mature ascomata are erumpent collectively through a disc of a compact tomentum that develops into a stroma. With age or drought the tomentum may gradually disappear or be replaced by a compact KOH- stroma that may incorporate cells of the surrounding bark.

The dark yellow-brown or golden-brown tomentum, clavate asci and hyaline, non-septate, ascospores are similar to those of *J*. *aurea*, *J*. *stachybotryoides* A.I. Romero & Samuels and *Calosphaeria aurata* Nitschke. Both *Jattaea* species differ clearly from *J*. *ribicola* by shorter asci and in ascospore size. In *J*. *aurea* ascospores are longer (7.0–)8.0–9.0 × (1.5–)2.0 μm, suballantoid to oblong and slightly curved [8], *J*. *stachybotryoides* possesses smaller, ellipsoidal-oblong ascospores, 3.0–6.0 × 1.5–2.0 μm [58]. Ascomata of both *Jattaea* species do not form circular groups under the periderm, they grow solitarily or are gregarious on decorticated wood.

In the holotype collection of *C*. *aurata* (Germany. Nordhein-Westfalen: Münsterland, near Münster-Angelmodde, decaying wood of *Alnus* sp., Oct 1865, T. Nitschke, B 700009123), ascomata were aggregated in loosened valsoid groups with decumbent, radially converging necks or grew solitarily on wood beneath the periderm, ascospores were longer than those of *J*. *ribicola*, (6.5–)7.0–8.5(–9.5) × 1.0–2.0 μm (mean ± SD = 7.8 ± 0.7 × 1.4 ± 0.2 μm). We could measure ascospores mostly within the asci, because only few were released. In the protologue [59] the size of ascospores is given as 8.0–10.0 × 1.5–2.0 μm. The tomentum of *C*. *aurata* is apparently of different origin as compared to *J*. *ribicola*. It is not formed by hyphae, but by small, bright yellow to golden angular cells releasing a yellow pigment in 3% KOH.

**Fig 11:**
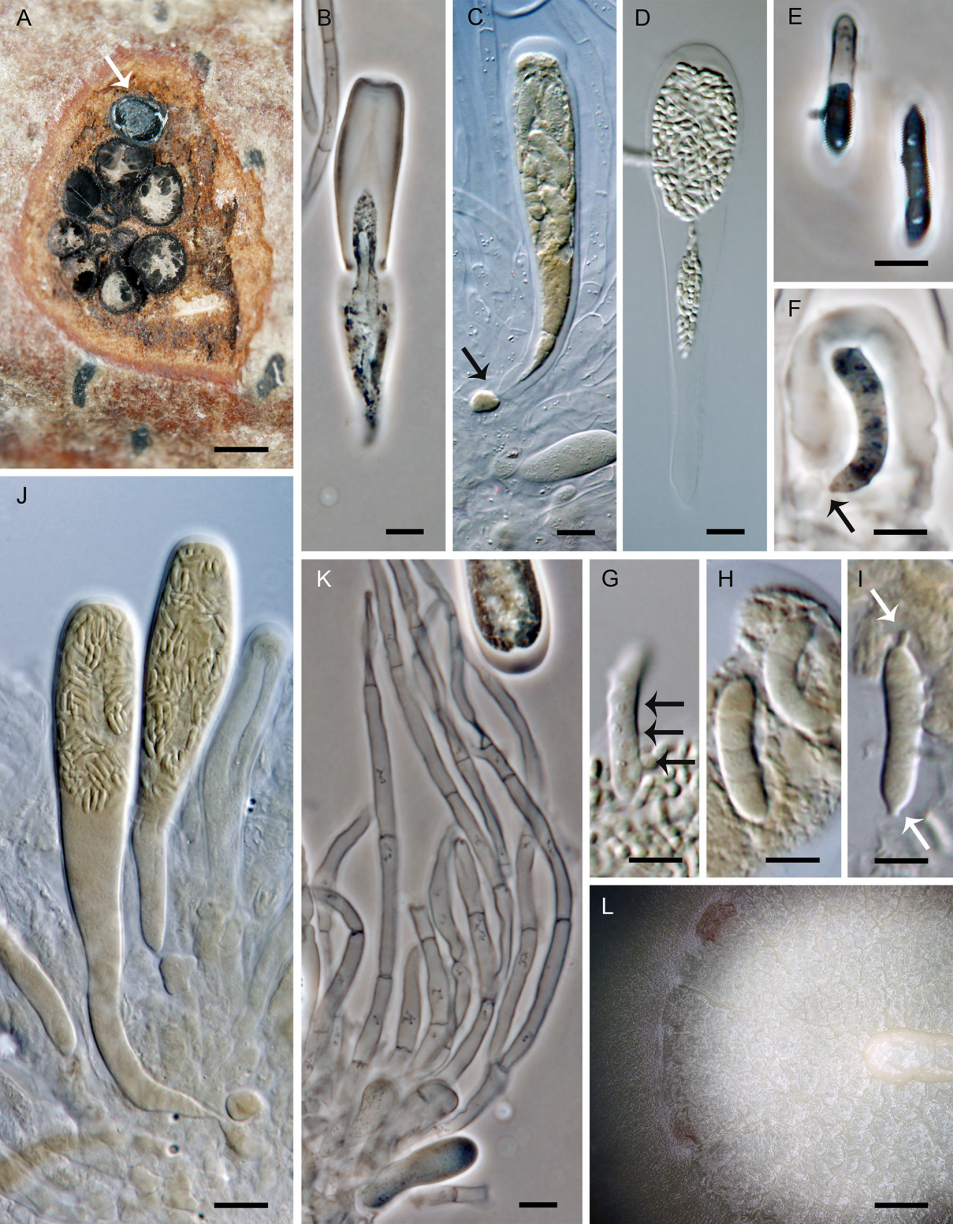
*Jattaea taediosa*, a lignicolous species with multiseptate ascospores producing ascoconidia. (A) Ascomata. (B) Ascus dehiscence. (C, D, J) Asci filled with numerous ascoconidia and eight ascospores. (E–I) Ascospores (arrows indicate either loci in the middle cells or short pegs in the terminal cells from which ascoconidia are produced). (K) Paraphyses. (L) Colony. DIC (C, D, J, G–I), PC (B, E, F, K), bar = 400 μm (A), 10 μm (B–D, J, K), 5 μm (E–I), 0.5 cm (L). C 69338 (A, C, J, H, I), PRM 934412 (B, D, E, F, K; L on MLA 21 d).

***Jattaea taediosa*.** (Sacc.) Réblová & Jaklitsch, comb. nov. (Fig 11A–11L)

[urn:lsid:indexfungorum.org:names:814420]

Basionym. *Calosphaeria taediosa* Sacc., Michelia 1: 368. 1878.

= *Calosphaeria cryptospora* Munk, Dansk Bot. Arkiv 17: 279. 1957.

*Ascomata* non-stromatic, growing on decaying wood beneath the periderm solitarily or in loose circular groups of 3–8 individuals around old ascomata of *Cryptosporella suffusa*; venter 400−450 μm diam, 400−550 μm high, subglobose, dark brown, upright or lying partly horizontally to the wood, slightly flattened laterally, glabrous, neck central, 100−120 μm wide, up to 500 μm long, cylindrical, partly parallel to the substratum, rounded at the top. *Ostiole* periphysate. *Ascomatal wall* 30−35 μm thick, leathery to fragile, two-layered. Outer layer consisting of brown *textura prismatica*, towards the interior grading into several layers of thin-walled subhyaline to hyaline flattened cells. *Paraphyses* persistent, septate, sparsely branched, hyaline, ca. 3.5–4.5 μm wide, slightly tapering towards the tip, longer than the asci. *Ascogenous hyphae* discrete, inconspicuous, short, with obpyriform to obovoid 4.0–4.5 wide, 6.5–7.5 μm long cells. *Asci* (70–)74–90(–110) × (14–)15–19 (–21) μm (mean ± SD = 84.2 ± 11.4 × 17.0 ± 1.8 μm), with sporiferous part (36–)40–66 μm long (mean ± SD = 49.8 ± 8.7 μm), broadly clavate, stipitate, tapering towards the base; apex obtuse, broadly rounded to truncate, thick-walled, conspicuously thickened to 1.5–5.5 μm, lacking a distinct discharge mechanism. Asci floating freely in the centrum upon maturation, with a bristle-like appendage at the base, 8-spored, with numerous ascoconidia when mature. Ascospores 11–16 × (2.5–)3.0–3.5(–4.0) μm (mean ± SD = 14.4 ± 1.3 × 3.1 ± 0.5 μm), suballantoid to subcylindrical, curved, hyaline, smooth, 3–4-septate, arranged 2–3-seriately or in a fascicle. *Ascoconidia* 3.5–5.5 × 1.5–2.5 μm (mean ± SD = 4.4 ± 0.6 × 1.7 ± 0.3 μm), ellipsoidal to suballantoid, hyaline, smooth, formed from circular openings in each cell of the ascospores or on minute pegs at the terminal cells.

Characters in culture. Colonies on MLA reaching a radius of 15–20 mm after 21 d at 25 °C, circular, flat, waxy with a moist appearance, with entire to slightly filiform margins, developing several wrinkles and deep radial folds. Colony surface white (oac909); reverse ivory (oac816) in the centre, white (oac909) towards the margin. Mycelium consisting of sparsely branched, septate, hyaline hyphae 2.0–2.5 μm wide, smooth-walled, sterile.

Typification. No type specimen is preserved in PAD (R. Marcucci, pers. comm.), but it was illustrated by Saccardo. Holotype (iconotype). Illustration of *Calosphaeria taediosa* in Saccardo ([60]: tab. 479). Epitype, designated here. Austria. Vienna: 21st district, Marchfeldkanalweg, at Felix Slavik Straße, on twigs of *Alnus incana* associated with *Cryptosporella suffusa*, 10 Jun 2012, W. Jaklitsch (PRM 934412, epitype of *Calosphaeria taediosa*, culture lost).

Specimens examined. Austria. Kärnten: Ebenthal, Obermieger, Sabuatach, grid square 9452/2, on *Alnus incana*, 15 Oct 2000, W. Jaklitsch W.J. 1636 (WU 33580); St. Margareten im Rosental, Tumpfi, grid square 9452/4, on *Cryptosporella suffusa*/*Alnus glutinosa*, 21 Dec 1994, W. Jaklitsch W.J. 391 (WU 33579); Steiermark, Spital am Semmering, near Pfaffensattel, grid square 8460/2, on *Alnus viridis*, 15 Aug 2003, W. Jaklitsch W.J. 2329 (WU 33581); Vienna, 21st district, Marchfeldkanalweg, grid square 7764/2, on *Alnus incana*, 13 May 1999, W. Jaklitsch. Denmark. Syddanmark: Dalby Molle near Kolding, on twigs of *Alnus glutinosa* associated with *C*. *suffusa*, 30 Jan 1933, P. Larsen (C-F-69338, holotype of *Calosphaeria cryptospora*).

Comments. *Jattaea taediosa* is well-distinguishable from other *Jattaea* species by 3–4-septate suballantoid to subcylindrical ascospores producing numerous ascoconidia within the asci. The ascospores are usually obscured by the mass of ascoconidia. *Jattaea ceanothina* can be compared with *J*. *taediosa* by cylindrical-allantoid to oblong, slightly curved, hyaline, 3–7-septate ascospores of a comparable size, but they never produce ascoconidia [3, 8, 61].

Although there is no type material of *Calosphaeria taediosa* in PAD, our observations based on recently collected material on twigs of *Alnus* spp. in Austria match the original description and illustration in Saccardo [60] and Berlese [62]. Therefore, Saccardo’s illustration serves as iconotype ([61]: tab. 479), and *Calosphaeria taediosa* is epitypified with a recently collected specimen (PRM 934412). The living culture is no longer available. Based on the revision of the holotype of *Calosphaeria cryptospora* [1], we conclude that both taxa are conspecific and the latter taxon is synonymised with *J*. *taediosa*. Munk [1] could not determine the number of ascospores in the ascus; he described the asci as apparently polysporous or with very numerous subcylindrical ascospores that produce masses of allantoid ascoconidia, which entirely cover them.

**Fig 12:**
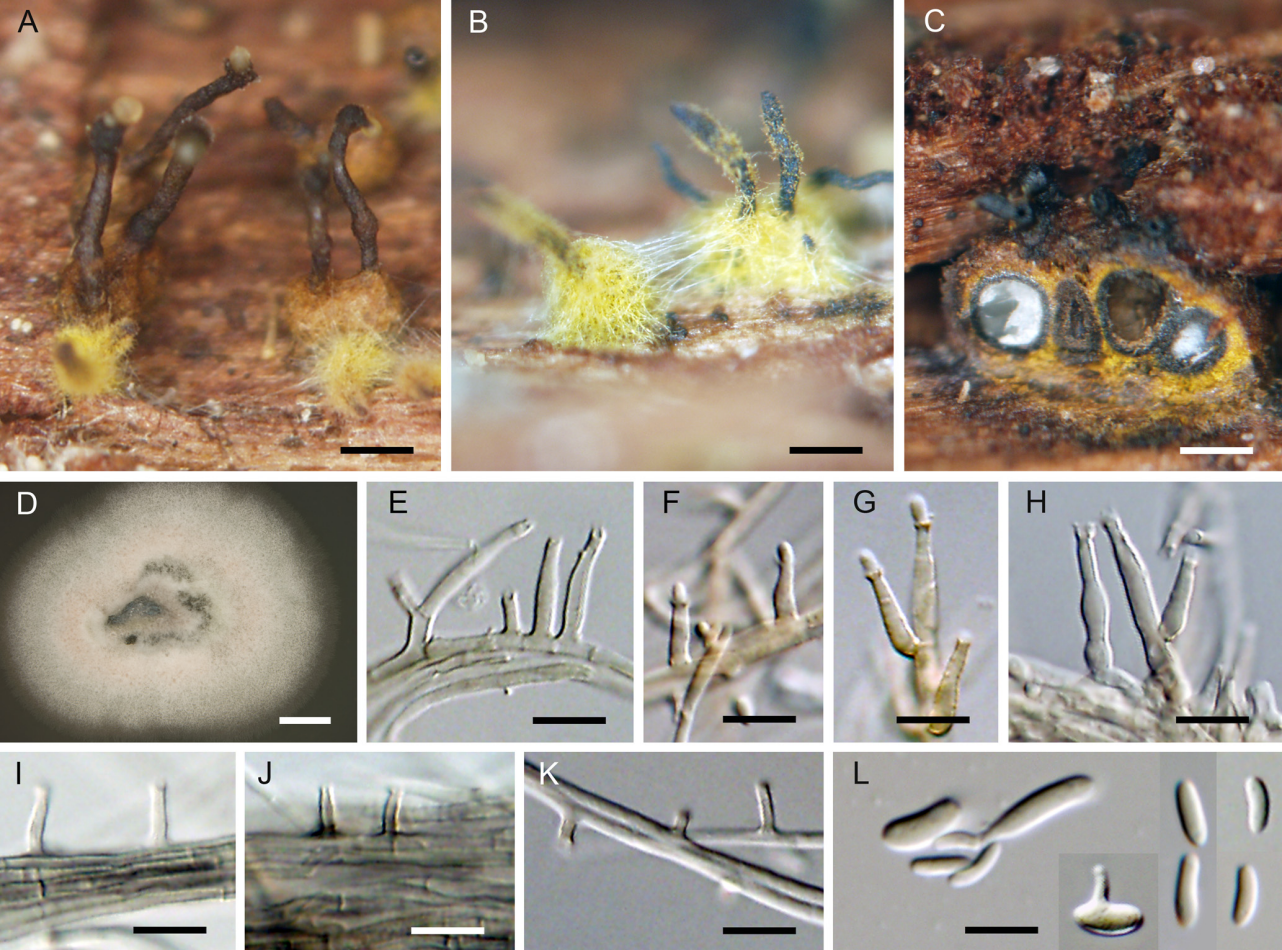
*Jattaea aurea*, a lignicolous species with a golden tomentum surrounding ascomata. (A–C) Ascomata. (D) Colony. (E–H) Phialides. (I–K) Adelophialides. (L) Conidia and two inflated conidia with a single phialidic opening. DIC (E–L), bar: 300 μm (A–C), 1 cm (D), 10 μm (E–K), 5 μm (L). J.P.P. 11242 (A–C), CBS 140209 (D–L on MLA 14 d).

*Jattaea aurea* Réblová & J. Fourn., Fung. Diver. 49: 175. 2011. (Fig 12A–12L)

Characters in culture. Colonies on MLA reaching a radius of 24–28 mm after 14 d at 25 °C, circular, flat, fluffy, dense, zonate, with entire margins. Aerial mycelium abundant, colony surface white (oac909) to ivory (oac816) in the centre, with zones of salmon pink (oac794) and beige (oac809) with grey-green undertones (oac830), white (oac909) to pale salmon pink (oac794) towards the margin, becoming pink-orange (oac764) at the margin with age; reverse white-green (oac893), white (oac909) towards the margin.

Mycelium consisting of branched, septate, hyaline to subhyaline hyphae, about 2.0 μm wide, smooth-walled. *Conidiophores* semi-macronematous, often reduced to a single conidiogenous cell, up to 20 μm long, 2.0–2.5 μm wide, hyaline to pale brown in mass, sometimes branched, 0–2-septate. *Phialides* discrete on aerial mycelium or densely aggregated on hyphae of substrate and aerial mycelium, 7.5–19 × (1.6–)2.5–3.0 μm (mean ± SD = 13.9 ± 5.5 × 2.5 ± 0.4 μm), cylindrical to elongate-ampulliform, often attenuated at the base, gradually tapering to 1.0–1.5 μm, hyaline, with a single conidiogenous locus, developing a terminal, slightly darker flaring collarette, 1.0–1.5 μm high, 1.5–2.0 μm wide, with a periclinal thickening; *adelophialides* 3.5–7.0 × 1.5–2.0 μm (mean ± SD = 5.8 ± 1.5 × 1.6 ± 0.2 μm), cylindrical, slightly tapering to 1.0–1.5 μm. *Conidia* 3.5–4.5(–4.9) × 1.5–2.0 μm (mean ± SD = 4.1 ± 0.5 × 1.6 ± 0.2) μm, hyaline, non-septate, ellipsoidal to suballantoid, often slightly curved, smooth-walled.

Specimen examined. France. Brittany: Morbihan, Sérent, La Tourbière de Kerfontaine, on decaying wood of *Myrica gale* L., 4 Dec 2011, Jean-Paul Priou *J*.*P*.*P*. *11242* (culture CBS 140209).

Comments. For a full description of the sexual morph see [8]. *Jattaea aurea* forms solitary ascomata or small groups of 2–3 individuals on decaying wood, or short rows of several ascomata around the edge of bark still attached to the wood (Fig 12A and 12B). Rarely circular groups of several aggregated ascomata are formed between the cortex and wood, with upright necks, tightly surrounded by the golden tomentum developing into a compact stromatic layer (Fig 12C). The brightly pigmented tomentum releasing pigment in 3% KOH also occurs in *J*. *stachybotryoides*, but it is golden-brown and this species differs from *J*. *aurea* by shorter ascospores and narrower asci [58].

Similar pigment is released into the agar medium and is present in vegetative hyphae in axenic culture. Green crystals of this pigment are well-visible in dehydrated mycelium in microscopic preparations.

**Fig 13:**
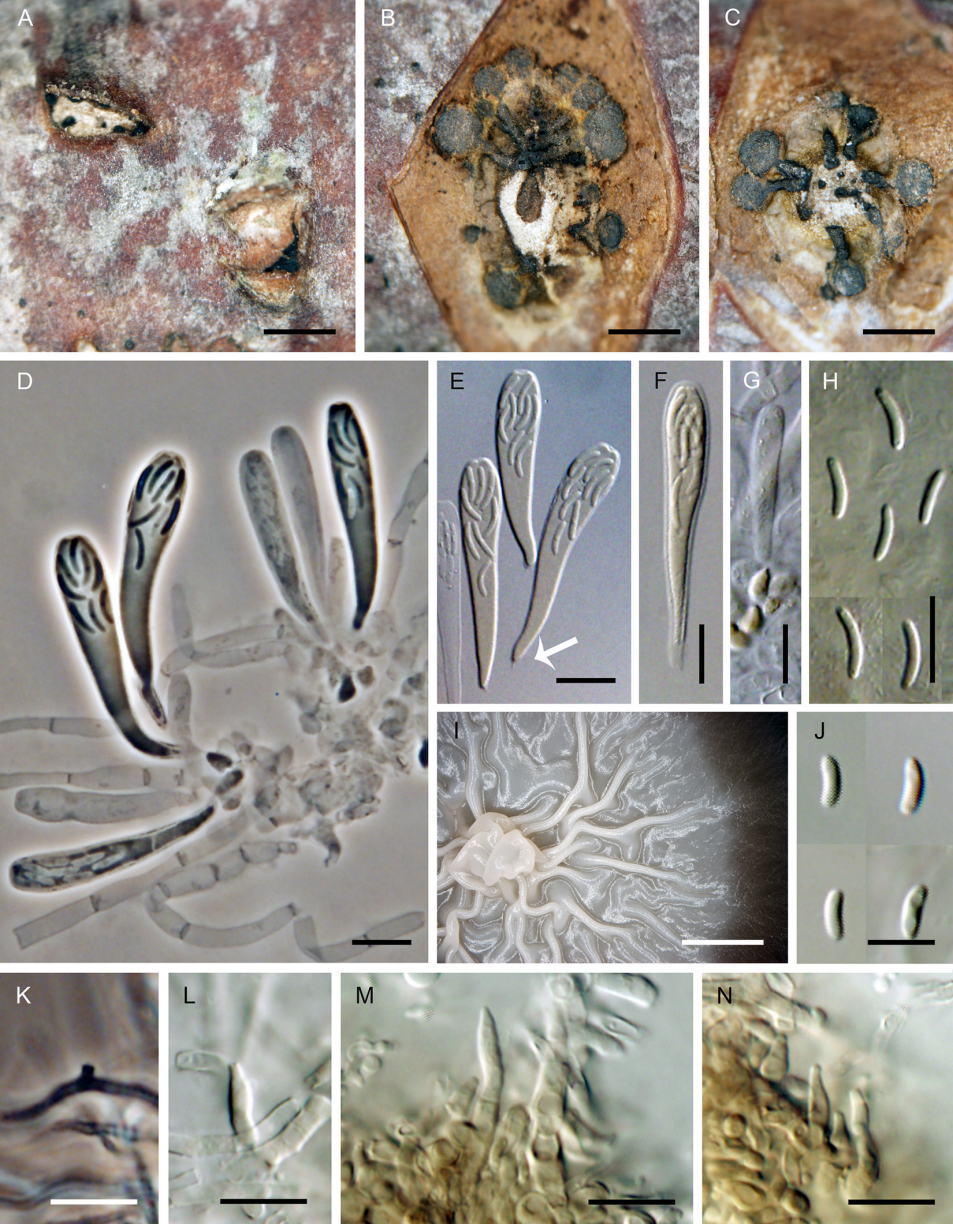
*Jattaea tumidula* on the host and in culture. (A–C) Ascomata. (D–G) Asci (arrow indicates a bristle-like appendage). (H) Ascospores. (I) Colony. (J) Conidia. (K) Adelophialide. (L–N) Phialides. DIC (E–H, J, L–N), PC (D, K), bar = 500 μm (A–C), 10 μm (D–H, K–N), 5 μm (J), 1 cm (I). PRM 934330 (A–M), CBS 140208 (H, J–M on MLA 14 d, I 21 d).

*Jattaea tumidula* (Sacc.) Réblová, Fung. Diver. 49: 186. 2011. (Fig 13A–13N)

≡ *Calosphaeria tumidula* Sacc., Atti Soc. Veneto-Trent. Sci. Nat. Padova 4: 77–100 (Fungi Ven. novi Ser. 4: 20) 1875. Fungi Italici Autographice delineati. Fascs. 9–12, Tab. 474. 1878.

≡ *Togninia minima* var. *tumidula* (Sacc.) Berl., Icon. Fung. 3: 11. 1900.

= *Ceratostomella mali* Ellis & Everh., Proc. Acad. Nat. Sci. Philad. 42: 225. 1890.

Characters in culture. Colonies on MLA reaching a radius of 20–23 mm after 14 d at 25 °C, circular, flat, smooth, glistening, deeply wrinkled and folded, with entire margins. Aerial mycelium sparse at the centre of the colony, colony surface white (oac909) to ivory (oac816); reverse beige pink (oac815) in the centre, becoming ivory (oac816) to inconspicuous towards the margin. Mycelium consisting of branched, septate, subhyaline hyphae, 1.5–2.0 μm wide, smooth-walled. *Conidiophores* micronematous, reduced to conidiogenous cells. *Phialides* 6.0–8.0 × 2.5–3.5 μm (mean ± SD = 7.3 ± 0.9 × 2.9 ± 0.4 μm), cylindrical to elongate-ampulliform, hyaline, gradually tapering to 1.0–1.5 μm; *adelophialides* 1.5–2.5 × 1.5–2.0 μm (mean ± SD = 1.8 ± 0.6 × 1.7 ± 0.5 μm), subcylindrical, tapering to ca. 1.5 μm, hyaline; collarette shallow, flaring about 2.5 μm wide. *Conidia* 4.0–5.5(–6.5) × 1.0–1.2 μm (mean ± SD = 4.9 ± 0.7 × 1.1 ± 0.3) μm, hyaline, non-septate, suballantoid, smooth-walled.

Typification. Holotype. Italy. Treviso: Cansigio, branch of *Fagus sylvatica*, Saccardo (PAD, holotype of *Calosphaeria tumidula*). Epitype, designated here. France. Midi-Pyreneés: Ariège, Montségur, road D9 ca. 1 km from the village, banks of the Le Lasset brook, on decaying branch of *Betula verrucosa*, 1 Oct 2013, M. Réblová M.R. 3729 (PRM 934330, epitype of *Calosphaeria tumidula*, culture ex-epitype CBS 140208).

Comments. For full description of the sexual morph refer to [6, 8], synonymy according to the latter author. In the type material of *J*. *tumidula* (PAD) collected by Saccardo [61] on *Fagus sylvatica* and in the recent collection (PRM 934330), the ascomata are growing in loose valsoid groups between periderm and wood, with necks partly parallel to the substratum, then ascending, converging radially and piercing the periderm in small circular fissures causing raised and blister-like areas on the bark. The ascomata in the collection PRM 934330 were flattened laterally and arranged around an old stromatic ascomycete (Fig 13A–13C). In the type material of *Ceratostomella mali* Ellis & Everh. [63], a synonym of *J*. *tumidula* [8], collected on *Malus* sp. (NY 00911859), the ascomata were scattered with venters immersed in decaying wood and only necks emerging.

**Fig 14:**
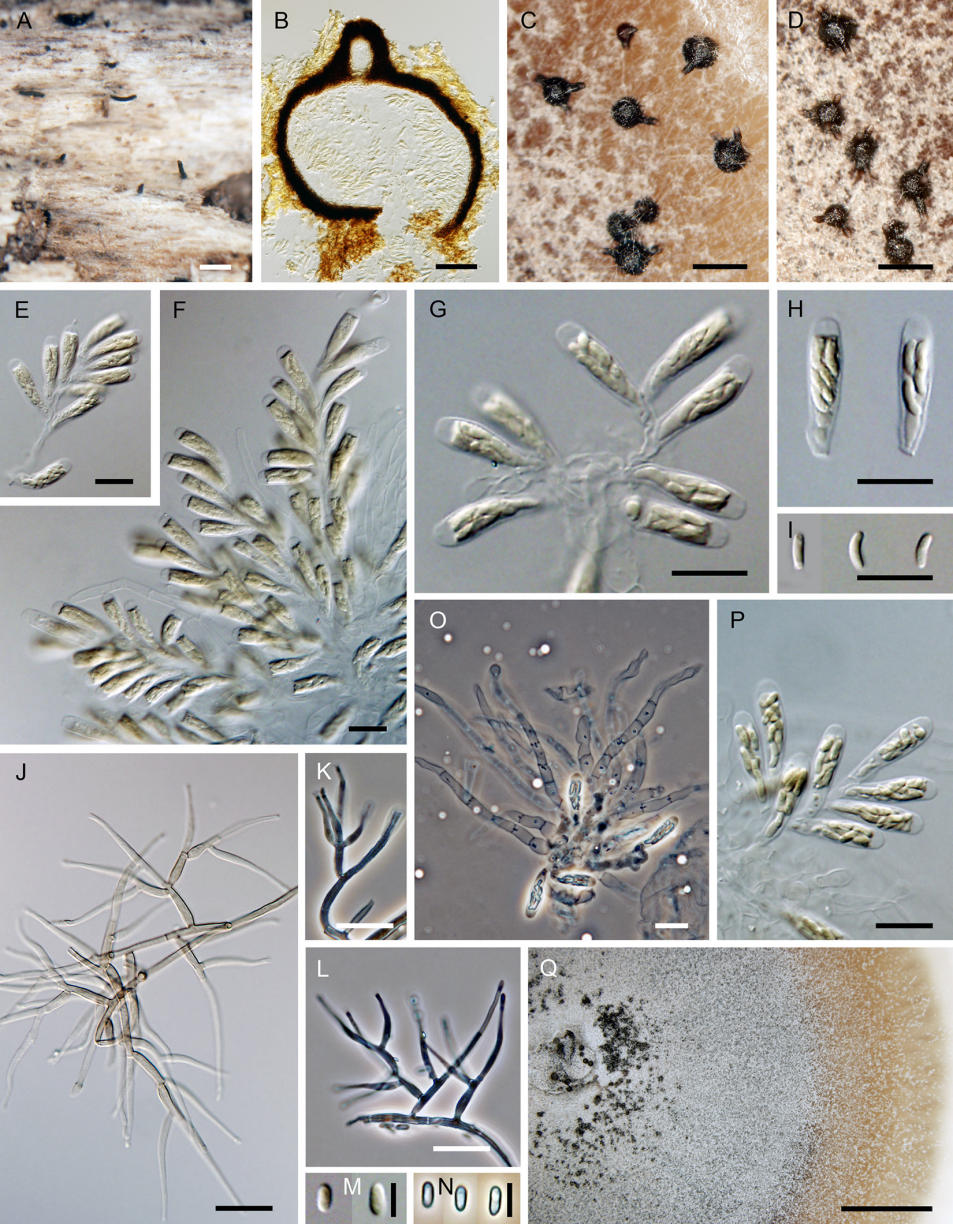
*Phaeoacremonium cinereum* on the host and in culture. (A) Ascomata on the host. (B) Vertical section of the ascoma. (C, D) Ascomata *in vitro*. (E–H) Asci. (I) Ascospores. (J–L) Conidiophores with phialides. (M, N) Conidia. (O, P) Asci with paraphyses *in vitro*. (Q) Colony. DIC (E–J, M, P), PC (K, L, N, O), bar = 500 μm (A, C, D), 50 μm B), 10 μm (E–L, O, P), 5 μm (M, N), 0.5 cm (Q). PRM 934331 (A, B, E–I), CBS 138685 (C, D, J–Q on MLA 21).

*Phaeoacremonium cinereum* Gramaje et al., Mycologia 101: 924. 2009. (Fig 14A–14Q)

*Ascomata* non-stromatic, immersed in decaying wood, only necks emerging, scattered; venter 250–300 μm diam, 250–350 μm high, subglobose, dark brown; neck central, 80−90 μm wide, 200–500 μm long, cylindrical, straight or slightly flexuous, rounded at the apex. *Ostiole* periphysate. *Ascomatal wall* 15–20 μm thick, leathery to fragile, two-layered. Outer layer consisting of brown *textura prismatica*, towards the interior grading into several layers of thin-walled subhyaline to hyaline flattened cells. *Paraphyses* persistent, septate, slightly inflated between the septa near their base, hyaline, 4.5–5.5 μm wide near the base, about 2.5 μm wide at the apex, longer than the asci. *Ascogenous hyphae* elongated, sparsely branched, with asci produced from croziers in spicate formation. *Asci* 18–19.5(–22) × 4.5–5.0(–5.5) μm (mean ± SD = 18.8 ± 1.3 × 5.1 ± 0.3 μm), L/W 3.7:1, clavate, apex broadly rounded to obtuse, thickened to 2.0–3.0 μm, lacking a distinct discharge mechanism. Asci floating freely in the centrum upon maturation, 8-spored. *Ascospores* 5.0–5.5(–6.0) × 1.5–2.0 μm (mean ± SD = 5.4 ± 0.3 × 1.6 ± 0.2 μm), suballantoid to oblong with rounded ends, hyaline, smooth, non-septate, arranged 2–3-seriately.

Characters in culture. Colonies on MLA reaching a radius of 28–35 mm after 14 d at 25 °C, circular, flat, felty, dense, zonate, with entire margins. Aerial mycelium abundant, colony surface pale brown-grey (oac774) at the centre, paler (oac809) towards the margin; reverse dark brown (oac768) at the centre. Mycelium consisting of branched, septate, pale brown hyphae, 2.5–3.0 μm wide, solitary or in strands, smooth-walled. *Conidiophores* semi-macronematous, arising from aerial and submerged hyphae, straight or flexuous, septate, branched, bearing one to several phialides, sometimes reduced to conidiogenous cells, attenuated at the base of the basal cell. *Phialides* terminal or lateral, monophialidic or with 1–2 lateral phialidic openings, smooth, collarettes hyaline, flaring, 1.5–2.5 μm long, 1.5–2.5 μm wide; type I phialides subcylindrical to navicular, attenuated at the base, 5.0–8.0 × 2.0–3.5 (mean ± SD = 7.8 ± 0.8 × 3.2 ± 0.8 μm); type II phialides subcylindrical to elongate-ampulliform attenuated at the base, 12–14.5 × 2.0–2.5 (mean ± SD = 23.2 ± 0.9 × 2.2 ± 0.3 μm); type III phialides subcylindrical to subulate, 15.5–25(–27) × 2.0–3.0(–3.5) (mean ± SD = 19.8 ± 3.5 × 2.5 ± 0.5 μm). *Conidia* (3.5–)4.0–5.0 × 2.0–2.5 (mean ± SD = 4.2 ± 0.4 × 2.1 ± 0.4 μm), ellipsoidal to oblong-ellipsoidal, non-septate, hyaline, smooth.

Specimen examined. France. Midi-Pyreneés: Ariège, Rimont, road D18 ca. 1.5 km from the village, banks of the Le Baup brook, on branch of *Betula verrucosa*, 1 Oct 2013, M. Réblová M.R. 3796 (PRM 934331, culture CBS 138685).

Comments. *Phaeoacremonium cinereum* was isolated from wood of *Vitis vinifera* showing symptoms of esca and Petri disease in Iran and Spain [18]. Its sexual morph was encountered for the first time on decaying deciduous wood in southern France and the ascospores were isolated in axenic culture. The strain CBS 138685, which was derived from the multi-ascospore isolate, is probably heterothallic; fertile ascomata identical to those *in vivo* were formed on PCA (Fig 14B and 14C) and MLA (Fig 14P) on the surface of the colony. The ascospores formed *in vitro* were slightly shorter than those from ascomata growing on wood.

Morphologically this species is similar to other members of the *P*. *parasiticum* group, but it is easily distinguished by grey pigmentation of the colonies on MEA [18] and conspicuous, subulate type III phialides. Based on shape and size of ascospores, it can be compared to *P*. *rubrigenum* W. Gams, Crous & M.J. Wingf. and *P*. *vibratile*.

### Genera accepted in the Calosphaeriales

The abolishment of dual nomenclature for pleomorphic fungi and amendment of Art. 59 in the International Code of Nomenclature for algae, fungi and plants [64–66] gave the sexual and asexual names of fungi an equal status, basically competing only by priority of publication. In the Calosphaeriales three asexual genera were introduced and linked with older names of their sexual morphs based on molecular data and cultivation experiments, i.e. *Calosphaeriophora* and *Phaeocrella* [4] and *Pleurostomophora* [5]. According to the new rules, *Calosphaeria* Tul. & C. Tul. and *Pleurostoma* Tul. & C. Tul. [67] have priority over *Calosphaeriophora* and *Pleurostomophora*. Although *Togniniella* and *Phaeocrella* were introduced in the same study [4], the name for the sexual morph is selected for this genus following the principle of priority.

Five genera, including *Flabellascus* described above, are accepted in the Calosphaeriales.

***Calosphaeria*** Tul. & C. Tul., Select. Fung. Carpol. (Paris) 2: 108. 1863.

= *Calosphaeriophora* Réblová, L. Mostert, W. Gams & Crous, Stud. Mycol. 50: 542. 2004. [Type species: *Calosphaeriophora pulchella* (Pers.) Réblová, L. Mostert, W. Gams & Crous, Stud. Mycol. 50: 542. 2004. = *Calosphaeria pulchella* (Pers.) J. Schröt., in Cohn, Krypt.-Fl. Schlesien (Breslau) 3.2(4): 451. 1897.]

Type species. *Calosphaeria princeps* Tul. & C. Tul., Select. Fung. Carpol. (Paris) 2: 109. 1863.

***Jattaea*** Berl., Icon. Fung. 3: 6. 1900.

= *Wegelina* Berl., Icon. Fung. 3: 8. 1900.

= *Phragmocalosphaeria* Petr., Annls. Mycol. 21: 109. 1923.

Type species. *Jattaea algeriensis* Berl., Icon. Fung. 3: 7. 1900.

= *Jattaea prunicola* Damm & Crous, Persoonia 20: 45. 2008.

***Togniniella*** Réblová, L. Mostert, W. Gams & Crous, Stud. Mycol. 50: 543. 2004.

= *Phaeocrella* Réblová, L. Mostert, W. Gams & Crous, Stud. Mycol. 50: 545. 2004.

Type species. *Togniniella acerosa* Réblová, L. Mostert, W. Gams & Crous, Stud. Mycol. 50: 545. 2004.

= *Phaeocrella acerosa* Réblová, L. Mostert, W. Gams & Crous, Stud. Mycol. 50: 545. 2004.

***Pleurostoma*** Tul. & C. Tul., Select. Fung. Carpol. (Paris) 2: 247. 1863.

= *Pleurostomophora* Vijaykr., L. Mostert, Jeewon, W. Gams, K.D. Hyde & Crous, Stud. Mycol. 50: 390. 2004. [Type species: *Pleurostomophora ootheca* Vijaykr., Jeewon & K.D. Hyde, Stud. Mycol. 50: 391. 2004. = *Pleurostoma ootheca* (Berk. & M.A. Curtis) M.E. Barr, Mycologia 77: 564. 1985.]

Type species. *Pleurostoma candollei* Tul. & C. Tul. (as *candollii*), Select. Fung. Carpol. (Paris) 2: 247. 1863.

### New combinations proposed in *Pleurostoma*.

***Pleurostoma ochraceum*.** (Mhmoud, Abd. Ahmed, Fahal, de Hoog & Sande) Réblová & Jaklitsch, comb. nov.

[urn:lsid:indexfungorum.org:names:814421]

Basionym. *Pleurostomophora ochracea* Mhmoud, Abd. Ahmed, Fahal, de Hoog & Sande, J. Clin. Microbiol. 50: 2990. 2012.

***Pleurostoma repens*.** (R.W. Davidson) Réblová & Jaklitsch, comb. nov.

[urn:lsid:indexfungorum.org:names:814422]

Basionym. *Cadophora repens* R.W. Davidson, J. Agric. Res. 50: 803. 1935.

≡ *Phialophora repens* (R.W. Davidson) Conant, Mycologia 29: 598. 1937.

≡ *Pleurostomophora repens* (R.W. Davidson) L. Mostert, W. Gams & Crous, Stud. Mycol. 50: 392. 2004.

***Pleurostoma richardsiae*.** (Nannf.) Réblová & Jaklitsch, comb. nov.

[urn:lsid:indexfungorum.org:names:814423]

Basionym. *Cadophora richardsiae* Nannf. apud Melin & Nannf., Svenska Skogsvårdsfören. Tidskr. 32: 421. 1934.

≡ *Phialophora richardsiae* (Nannf.) Conant, Mycologia 29: 598. 1937.

≡ *Pleurostomophora richardsiae* (Nannf.) L. Mostert, W. Gams & Crous, Stud. Mycol. 50: 392. 2004.

= *Cadophora brunnescens* R.W. Davidson, J. Agric. Res. 50: 803. 1935.

≡ *Phialophora brunnescens* (R.W. Davidson) Conant, Mycologia 29: 598. 1937.

### Key to genera of the Calosphaeriales

1. Asci polysporous, ascomata stipitate with a lateral papilla …………………… *Pleurostoma*

1. Asci 8-spored, ascomata non-stipitate with a long neck …………………………………… 2

        2 Ascospores non-septate, usually 0.5–1.0 μm wide; ascogenous hyphae elongated …. 3

        2 Ascospores septate or non-septate, 1.5–3.0 μm wide; ascogenous hyphae shorter …. 4

3. Phialides ampulliform, conidiophores dark brown, ending in a terminal phialide or a whorl of phialides, with several lateral phialides or a series of branches terminating in verticillate phialides ……………………………………………………………………… *Flabellascus*

3. Phialides subcylindrical, navicular to elongate-ampulliform, conidiophores pale brown to subhyaline, sparsely branched, whorls of phialides not formed ………………. *Togniniella*

        4. Ascomata arranged in large circular or oval formations or nests, often in several vertical levels on wood beneath the loosened periderm; asci clavate with a long, slender, gradually tapering stipe ………………………………………. *Calosphaeria*

        4. Ascomata solitary to gregarious or in small, loose valsoid groups on wood beneath the periderm, or immersed in decaying wood; asci oblong to clavate, narrowly rounded at the base or with a tapering stipe …………………………………. *Jattaea*

### Genera of uncertain taxonomic status morphologically similar to members of the Calosphaeriales

Calosphaeriales was founded as a polyphyletic group of phenotypically similar stromatic and non-stromatic perithecial ascomycetes classified into two families, the Calosphaeriaceae and Graphostromataceae [2, 3, 60], encompassing nine genera, viz. *Calosphaeria*, *Enchnoa* Fr., *Graphostroma* Piroz., *Jattaea*, *Pachytrype* Berl. ex M.E. Barr, J.D. Rogers & Y.M. Ju, *Pleurostoma*, *Romellia* Berl., *Scoptria* Nitschke and *Phaeoacremonium* (as *Togninia*). Twenty years later, the majority of these taxa were revised with the aid of molecular DNA data and confirmed to belong to morphologically similar groups that evolved from different phylogenetic lineages.

The Graphostromataceae based on *Graphostroma platystoma* (Schwein.) Piroz. with a *Nodulisporium*-like asexual morph was suggested to be included in the Xylariales [68]. Its new placement was later confirmed with molecular data [69], although *Graphostroma* may belong to the Xylariaceae. *Graphostroma* is characterised by flat widely effused stromata, clavate asci with an amyloid apical annulus, hyaline, non-septate, almost allantoid ascospores, absence of a typical calosphaeriaceous centrum, holoblastic denticulate conidiogenesis and occurrence on decaying wood of deciduous trees [70].

*Pachytrype* is a genus of stromatic ascomycetes with more or less rectangular to oblong, sessile asci with an apical ring and hyaline, non-septate, ellipsoidal to oblong ascospores, producing *Cytospora*-like asexual morph in axenic culture [60, 71]. Based on nuc28S rDNA sequences, *P*. *princeps* (Penz. & Sacc.) M.E. Barr, J.D. Rogers & Y.M. Ju, the type species, and *P*. *rimosa* F.A. Fernández et al., were placed in the Diaporthales [72].

*Phaeoacremonium* including *Romellia* as its generic synonym was excluded from the Calosphaeriales based on DNA sequences of nuclear ribosomal and protein-coding genes and morphological data and transferred to the Togniniaceae [4, 73]. This family is a well-established monophyletic group comprising species with a global distribution commonly isolated from rootstocks, stems and branches of diseased woody hosts, and also humans with phaeohyphomycosis [6, 14–16, 18, 27, 29, 74].

*Scoptria* was included in the Calosphaeriales along with its synonyms *Peroneutypella* Berl. and *Wegelina* Berl. [3]. Today, *Scoptria* and *Peroneutypella* are treated as synonyms of *Eutypella* (Nitschke) Sacc. of the Diatrypaceae [75], while *Wegelina* [76] remained in the Calosphaeriales as a generic synonym of *Jattaea* [8].

*Enchnoa* represents another genus attributed to the Calosphaeriales [3]. Later, based on a freshly collected material of *Enchnoa infernalis* (Kunze) Fuckel, Barr [3] re-evaluated its systematic placement and suggested that *Enchnoa* is better placed in the Nitschkiaceae of the Coronophorales based on the presence of a shallow Quellkorper near the ascoma apex [77].

In the Outline of Ascomycota [78] three other genera were placed in the Calosphaeriales with a reservation, viz. *Conidiotheca* Réblová & L. Mostert, *Kacosphaeria* Speg. and *Sulcatistroma* A.W. Ramaley. The monotypic genus *Conidiotheca* [73] was described for *Romellia tympanoides* M. E. Barr [3] and characterised by non-stromatic, papillate ascomata growing between cortex and wood, cylindrical-clavate asci arising from croziers, apically thickened, lacking a visible discharge mechanism and containing eight ellipsoidal to fusiform, transversely and longitudinally septate ascospores producing numerous ascoconidia within the asci. The DNA data of this fungus are not available. The persistent ascogenous hyphae with minute cells in the ascoma centrum and asci in spicate arrangement, a hallmark of the Calosphaeriales, were not observed. The genus is temporarily placed as incertae sedis within the Sordariomycetes.

*Kacosphaeria antarctica* Speg. is the type and only species of the genus described from a decaying branch of *Ribes magellanicum* Poir. in Patagonia, Argentina [79]. The type material is not available. According to the protologue, the fungus bears a strong resemblance to species of *Jattaea* in non-stromatic perithecial ascomata arranged in loose valsoid formations beneath the periderm, clavate, stipitate asci with a thickened apex and distinct sporiferous part, persistent paraphyses and allantoid, 1-septate, hyaline ascospores. Confirmation of the systematic position of *Kacosphaeria* and its presumed relationship with the Calosphaeriales will require recollection of the type species and subjecting it to DNA sequencing.

*Sulcatistroma nolinae* A.W. Ramaley is a stromatic perithecial ascomycete occurring on dead leaves of *Nolina micrantha* I.M. Johnst. (Asparagaceae) in southern United States [80]. Based on its hyaline to very pale brown ascomata immersed in a discrete stroma, clavate asci lacking an apical ring, allantoid, non-septate ascospores, paraphyses and phialidic conidiogenesis, the fungus was assigned to the Calosphaeriales and compared with stromatic members of the order. The typical calosphaeriaceous centrum is lacking, asci are basally narrowly rounded and do not arise in fascicles, spicate or palmate formations on ascogenous hyphae. Also the occurrence in dead leaves is atypical of members of the Calosphaeriales, which are all lignicolous. Until the DNA sequence data can prove or disprove the systematic placement of *Sulcatistroma*, we prefer to place the genus among taxa of uncertain taxonomic status in the Sordariomycetes.

## Discussion

### Calosphaeriaceae

The revised Calosphaeriales comprises five genera accommodated in two families, the Calosphaeriaceae [1] and Pleurostomataceae [4]. Members of the Calosphaeriaceae share a set of characters such as globose to subglobose dark ascomata with a central neck, hyaline, non-septate or one to several transversely septate, allantoid, suballantoid, oblong to subcylindrical ascospores, and 8-spored, clavate, stipitate asci tapering from the sporiferous part downwards, with a bristle-like appendage at the base, attached to ascogenous hyphae in characteristic formations or floating freely in the centrum upon maturation. The asci have a conspicuous, symmetrical thickening at the apex, which lacks a visible discharge mechanism. The fissitunicate ascus dehiscence, referred to as pseudofissitunicate in unitunicate ascomycetes by Eriksson [81], was observed in *Calosphaeria africana* [7]. Although all genera are non-stromatic, in some species of *Calosphaeria* and *Jattaea* the tomentum can develop into a thin, compact stromatic layer enclosing one or several ascomata.

Currently 85 species, varieties and synonyms are classified in *Calosphaeria* (Index Fungorum, www.indexfungorum.org). However, DNA sequence data are available for only two species of *Calosphaeria* s. str. Delimitation of *Calosphaeria* from morphologically similar but phylogenetically distantly related fungi entailed an understanding of life history, discovery of new asexual morphs and revision of some subtle morphological characters, especially those of asci and the ascogenous system. *Calosphaeria* s. str. is distinct from morphologically similar species having asexual morphs similar to *Sporothrix* Hektoen & C.F. Perkins and *Ramichloridium* Stahel ex de Hoog, e.g. *Calosphaeria barbirostris* (Dufour) Ellis & Everh., *C*. *dryina* (Berk. & Broome) Nitschke and *C*. *fagi* Samuels & Cand. This was recently corroborated by molecular DNA and morphological data and *in vitro* studies leading to their exclusion from the Calosphaeriales and the description of *Barbatosphaeria* Réblová with closest relatives in the Ophiostomatales [82–84]. Although several other *Calosphaeria* species were historically transferred to the Ceratostomataceae, Gnomoniaceae, Nitschkiaceae, Togniniaceae, Valsaceae or Sordariomycetes incertae sedis (sensu Index Fungorum), the genus remains a heterogeneous assemblage of phenotypically similar taxa and a taxonomic revision is still required.

*Jattaea* was recently revised and 14 species were accepted in the genus along with *Phragmocalosphaeria* Petr. and *Wegelina* as generic synonyms [8]. Two new species and one new combination in *Jattaea* are introduced in this study. The morphological delimitation of *Calosphaeria* from *Jattaea* is rather narrow and is complicated by similar morphology of their asci, ascogenous hyphae, ascospores and paraphyses. The ascomata of *Calosphaeria* are usually arranged in dense circular or oval formations, often in several vertical levels beneath the loosened periderm with long radially converging necks partly running parallel to the wood. The ascomata of *Jattaea* occur usually solitarily, scattered or in small irregular to valsoid groups on wood beneath the periderm, around old fungal stromata or margins of the peeled bark or are rarely immersed in decaying wood. Some species, however, have ascomata arranged in 1–2 vertical levels and in larger groups similar to *Calosphaeria*. The asci in *Jattaea* are oblong-clavate to clavate, short- to long-stipitate, while asci of *Calosphaeria* species tend to be mostly clavate with a long slender stipe. Their asexual morphs are morphologically similar dematiaceous phialidic hyphomycetes. Although the difference between *Calosphaeria* and *Jattaea* using morphological characters is narrow, it is remarkable at the RNA structural level of ITS rDNA, especially of ITS1.

Six unidentified strains of *Jattaea* characterised only by their ITS, nuc28S and β-tubulin sequences available in GenBank were included in our phylogeny. Strains labelled as *Jattaea* sp. 1 (strain HNDC06) and *Jattaea* sp. 2 (YNDC19, YNDC23) are endophytes isolated from Dragon´s blood samples of *Dracaena* spp. in China [85]. These three strains were preliminarily identified as *J*. *algeriensis*, but with more concentrated taxon sampling and study of the structural elements of ITS1 they represent two different species (Fig 1). However, their wild type and colony characters are unknown. Three other strains originate in South Africa [57]. *Jattaea* sp. 3 (CBS 122684, CMW 22119) from twig litter of *Protea* sp. and *Jattaea* sp. 4 (CBS 122685) collected on twig litter of *Leucospermum* sp. were originally listed to belong to a single species of *Togninia* sp. The description [57], however, was based on another isolate CBS H-20073, of which no DNA sequences are available. We could not locate herbarium material of any of these three strains. Based on molecular data these isolates represent at least two distinct species related to *J*. *aphanospora*, *J*. *discreta* and *J*. *taediosa*.

*Flabellascus* and *Togniniella* look virtually like miniature versions of *Calosphaeria* and *Jattaea*. They differ from the two latter genera by arrangement of ascomata on the host; they do not form any kind of valsoid or large circular groups beneath the periderm. They also differ by elongated and branched ascogenous hyphae. The ascospores of *Flabellascus* and *Togniniella* are generally shorter and narrower (0.5–1.0 μm wide) than those of *Calosphaeria* and *Jattaea* (1.5–3.0 μm wide). Their paraphyses are conspicuously inflated between the septa, wider near the base and gradually tapering, while paraphyses of *Calosphaeria* and *Jattaea* are mostly cylindrical, longer, not constricted or only slightly constricted at the septa.

*Flabellascus* bears a strong resemblance to *Togniniella*. Both genera are monotypic, characterised by long-necked ascomata scattered to gregarious, immersed in decaying wood, apically thickened clavate asci in spicate or fan-shaped formations, minute allantoid to suballantoid, non-septate ascospores and inflated, septate paraphyses. *Togniniella acerosa* differs from *F*. *tenuirostris* by slightly shorter and narrower asci (18–21(–22) × 3.0–4.0 μm, L/W 6:1), and narrower ascospores (only up to 0.5 μm wide) [4]. The main difference between *Togniniella* and *Flabellascus* lies in the characters of conidiophores, phialides and partly conidia formed *in vitro*. In the absence of the asexual morph, which has never been observed on the natural host, the distinction of the two genera is challenging.

All three strains of *T*. *acerosa* including the ex-type strain form a strongly supported monophyletic clade in our phylogeny (Fig 1). They originate in New Zealand and were collected in three different localities on decaying wood of *Nothofagus* sp. and other deciduous trees on Southern Island. *Ceratostomella microspora* Ellis & Everh. was described for a miniature specimen collected on decayed beech wood in North America [56]. This fungus did not match the recently emended generic concept of *Ceratostomella* Sacc. [86], it was later identified as *T*. *acerosa* Réblová et al. [4] and a new combination was proposed in the genus [8]. The slight difference in ascus and ascospore sizes between *T*. *acerosa* (PDD 81431, dimensions for asci and ascospores given above) [4] and *C*. *microspora* holotype (NY 00911861: asci (18.5–)19–22(–24.2) × 3.5–4.5 μm and ascospores 3.0–4.0 × 0.5–1.0 μm), was considered to be due to intraspecific variability. That the combination might have been premature became obvious, when six collections of a fungus strongly resembling *Togniniella* were made on *Fagus sylvatica* and *Quercus cerris* in two different localities in the Czech Republic. These specimens were also compared with two morphologically similar collections from North America, from Canada (DAOM 136897) and United States of America (holotype of *C*. *microspora*, NY 00911861).

Based on results of the five-gene analysis of morphologically similar strains originating in Northern and Southern hemispheres and their distinction into two strongly supported clades (Fig 1), we introduced *Flabellascus* for a *Togniniella*-like specimen from the Northern hemisphere (Europe). Another evidence that the strains from two different geographical zones represent two separate groups at the genus level was corroborated by computational analysis of the 2D structure of ITS and comparison of available ITS sequences of members of the Calosphaeriales. The strains of *T*. *acerosa* and *F*. *tenuirostris* showed significant genetic heterogeneity and a difference in the length of primary ITS1 sequences and in the topology of the 2D predicted model of ITS1. As the asexual morph of *C*. *microspora* is unknown, we prefer to avoid proposing a new combination for *C*. *microspora* in *Flabellascus* or accepting its former combination in *Togniniella* [8] without fresh material and DNA sequence data. However, additional taxon sampling will be necessary to support the theory that *Flabellascus* and *Togniniella* are geographically disconnected.

### Pleurostomataceae

**Fig 15:**
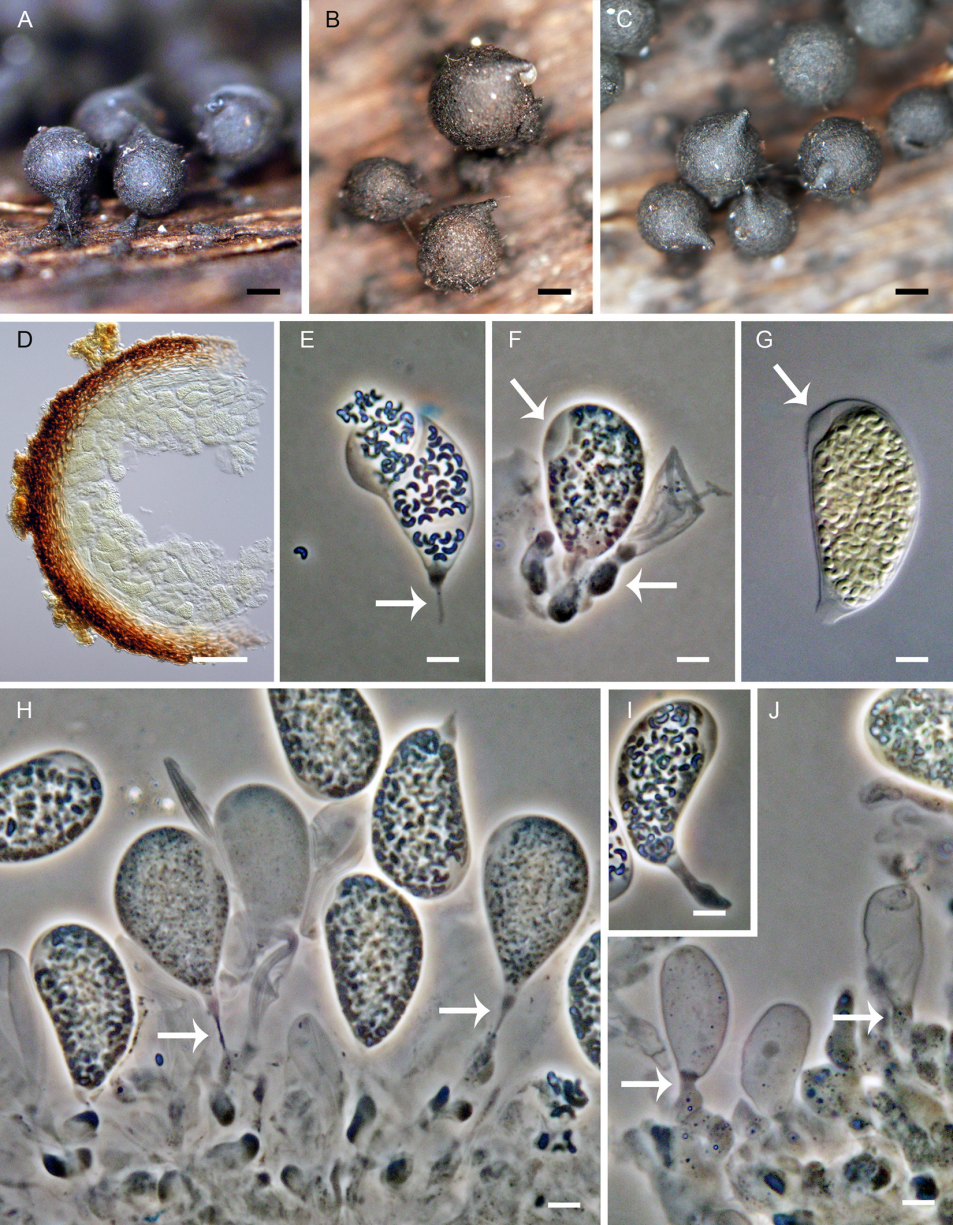
Two lignicolous species of *Pleurostoma* with stipitate ascomata. (A–C) *Pleurostoma candollei*: Ascomata. (D–J) *Pleurostoma ootheca*: (D) Vertical section of the ascoma. (E, H) Polysporous ascus with a bristle-like appendage. (F, I, J) Polysporous asci (arrows point at the robust stipes attached to cells on ascogenous hyphae and indicate thickening at the apex). (G) Polysporous ascus with a conspicuous thickening (arrow points at the asymmetrically thickened apex). DIC (D, G), PC (E, F, H–J), bar = 100 μm (A–C), 50 μm (D), 5 μm (E–J). J.F. 06057 (A, B), M.R. 3559 (C), K 122386 holotype of *P*. *ootheca* (D, G), K 122385 (E, F, H–J).

Pleurostomataceae is a monotypic family introduced for *Pleurostoma* [4]. It is well-distinguishable from other members of the Calosphaeriales by unique sexual morphological characters and structural characters of the ITS1. *Pleurostoma* is characterised [67] by globose to subglobose stipitate ascomata with 1–3 laterally positioned papillae, early deliquescing paraphyses, hyaline, allantoid, non-septate ascospores and polysporous, oval, stipitate asci (Fig 15A–15J). Unlike *Calosphaeria*, *Jattaea*, *Flabellascus* and *Togniniella*, the ascomata of *Pleurostoma* are not formed beneath the periderm and do not seem to occur near other ascomycetes. The ascomata are usually densely aggregated on decorticated decaying wood, forming extensive dark colonies. The ascal apex is asymmetrical; it is conspicuously thickened on the upper edge of the concave side and the sporiferous part is slightly invaginated (Fig 15F and 15G). The formation of minute cells on ascogenous hyphae is not as pronounced as it is in members of the Calosphaeriaceae.

Two sexually reproducing species are currently accommodated in *Pleurostoma*, viz. *P*. *candollei* Tul. & C. Tul., the type species, and *P*. *ootheca* (Berk. & M.A. Curtis) M.E. Barr. *Pleurostomophora* [5] was introduced for the asexual morph linked with *P*. *ootheca* and two other morphologically similar *Phialophora* species, *P*. *repens* (R.W. Davidson) Conant and *P*. *richardsiae* (Nannf.) Conant, both isolated from woody plants, soil or sewage [87]. The two latter species were also identified as etiological agents causing subcutaneous phaeohyphomycosis in humans [10–12]. *Pleurostomophora ochracea* Mhmoud et al. was introduced recently for a pathogenic fungus causing phaeohyphomycosis and a true mycetoma in humans [13]. These three asexual species were transferred to *Pleurostoma* in this study.

### Asexual morphs linked to the Calosphaeriales

The sexual-asexual connections of all five genera of the Calosphaeriales were established experimentally. The asexual morphs linked to *Calosphaeria*, *Jattaea* and *Pleurostoma* are reduced phialidic hyphomycetes similar to *Phialophora* and characterised by semi-macronematous, hyaline, subhyaline to pale yellow-brown conidiophores often reduced to conidiogenous cells such as phialides or adelophialides, i.e. single conidiogenous cells without a basal septum [88]. Phialides are hyaline, subhyaline or pale brown, sometimes pigmented in the apical region below the collarette; they are short-ampulliform to elongate-ampulliform to cylindrical, tapering, with a more or less conspicuous funnel-shaped collarette [4, 5, 7, 8].

The asexual morphs of *Calosphaeria* were experimentally proven for *C*. *africana* [7] and *C*. *pulchella* [4]. The asexual morphs of *Jattaea* have been experimentally established for nine of the 17 accepted species, i.e. *J*. *algeriensis*, *J*. *aphanospora*, *J*. *aurea*, *J*. *discreta*, *J*. *leucospermi* Marinc., M.J. Wingf. & Crous, *J*. *mookgoponga* Damm & Crous, *J*. *ribicola*, *J*. *taediosa* and *J*. *tumidula* ([7, 8,] this study).

The life history of *Pleurostoma* has been experimentally verified only for *P*. *ootheca*, while the asexual morph of *P*. *candollei* is unknown. Although an asexually reproducing fungus occurring on the host near ascomata was described and illustrated in the protologue of *P*. *candollei* [67], its interpretation is difficult. The original illustration ([67]: tab. XXVIII Figs 1–3) exhibits sporodochia with an outer palisade of hyaline conidiophores and numerous hyaline conidia; the conidiophores (as spermatia) are described as branched, conidia curved, approximately 3 μm long.

The conidiophores of *Flabellascus* and *Togniniella* are macronematous, adelophialides occur rarely. *Togniniella* possesses simply branched, pale brown conidiophores morphologically similar to *Phaeoacremonium*, though more regularly branched with constriction at the septa ending in subcylindrical to elongate-ampulliform phialides that never occur in whorls, with a shallow flaring collarette, producing only one type of obovoid to reniform conidia [4]. On the other hand, whorls of hyaline to subhyaline ampulliform phialides arising on dark brown branches or directly on conidiophores are typical of *Flabellascus*. Phialides are often slightly curved in the neck and produce two types of hyaline non-septate conidia.

### Togniniales

The acceptance of *Phaeoacremonium* over *Togninia* and its protection against the latter name was recently proposed [29]. So far, thirteen sexual-asexual relationships in *Phaeoacremonium* have been experimentally verified (Fig 2). *Phaeoacremonium aquaticum* (D.M. Hu, L. Cai & K.D. Hyde) D. Gramaje, L. Mostert & Crous [89], *P*. *leptorrhynchum* [8] and *P*. *inconspicuum* [6] are only known as sexual morphs and their life histories are yet to be proven experimentally.

The molecular identification of *Phaeoacremonium* species has been facilitated by Restriction fragment length polymorphisms (RFLP) of the ITS region, PCR-RFLP markers from the ITS and the β-tubulin gene, ITS barcode sequences including design of ITS species-specific primers for selected *Phaeoacremonium* species, and sequencing of multiple nuclear loci such as calmodulin, actin and partial β-tubulin [6, 15, 16, 18, 90, 91, 92]. In the phylograms inferred from Maximum Parsimony (MP) analysis of actin-β-tubulin with unspecified coding and non-coding regions [18, 29] and from ML and BI analyses of ITS-actin-β-tubulin sequences but with different partitions applied to coding and non-coding regions (this study), *Phaeoacremonium* forms three major clades labelled as *P*. *minimum*, *P*. *parasiticum* and *P*. *sicilianum* on Fig 2. The MP and ML/BI analyses differ by the robustness of the inferred trees. While in the MP analysis *Phaeoacremonium* was shown as a strongly supported clade (97% MP BS) with *P*. *minimum* (99) and *P*. *parasiticum* (83) subclades, in the ML/BI phylogram these groupings obtained generally lesser support (Fig 2). *Phaeoacremonium sicilianum* was always positioned basal to all taxa. In the third scenario, when members of the closely related Calosphaeriales were excluded and analysis was performed under the same options, the *P*. *parasiticum* clade was not recovered and instead the four individual subclades were shown separately positioned on the tree. Such topology is consistent with that published earlier [15]. The analysed coding regions of actin (116 nt) and β-tubulin (311 nt) are rather short fragments containing much less information as compared to non-coding regions. In protein-coding genes there are constraints on possible substitutions, and when the regions are small this may not be covered appropriately by ML substitution models.

Morphological distinction of *Phaeoacremonium* species is complicated by a relatively low degree of variability expressed in the characters of conidia, phialides and conidiophores, except for the two species *P*. *parasiticum* (Ajello, Georg & C.J.K. Wang) W. Gams, Crous & M.J. Wingf. and *P*. *inflatipes* W. Gams, Crous & M.J. Wingf., which can be readily identified by long and extensively branched conidiophores. The sexual morphs are morphologically also similar and the sizes of their asci and ascospores frequently overlap. The key to *Phaeoacremonium* species published by Mostert et al. [6] is based primarily on cultural characteristics such as growth, colony colour and optimum growth temperature, sometimes accompanied by morphological traits. Although the distribution of morphological characters of conidia and types I, II and III phialides are random in the three major clades recovered in *Phaeoacremonium* and cannot be used for their delimitation, we show that these three phylogenetic groups can be characterised at the RNA structural level by distinct topologies of the predicted 2D models of ITS1, by ascospore shape and partly by ecology and known pathogenicity to humans (Fig 2).

Members of the *P*. *parasiticum* clade are linked with sexual morphs that always have allantoid to suballantoid to subcylindrical ascospores, i.e. *P*. *aquaticum* [89], *P*. *cinereum* (this study), *P*. *krajdenii*, *P*. *parasiticum* and *P*. *rubrigenum* [6], and *P*. *vibratile* [73]. On the other hand, sexual morphs associated with the *P*. *minimum* clade have always ellipsoidal-oblong to reniform ascospores, i.e. *P*. *africanum* (Damm, L. Mostert & Crous) D. Gramaje, L. Mostert & Crous and *P*. *griseo-olivaceum* (Damm, L. Mostert & Crous) D. Gramaje, L. Mostert & Crous [15], *P*. *fraxinopennsylvanicum* and *P*. *novae-zealandiae* [28], *P*. *minimum* [14], *P*. *argentinense* L. Mostert, W. Gams & Crous, *P*. *austroafricanum* L. Mostert, W. Gams & Crous and *P*. *viticola* J. Dupont [6]. *Phaeoacremonium sicilianum* is known only as asexual morph [16].

Although species of the *P*. *parasiticum* clade were widely isolated from wood of *Vitis vinifera*, *Prunus* spp. and other trees, seldom from soil and arthropods, seven species were also reported from human subcutaneous infections, and additional three species are known solely as human pathogens (Fig 2). *Phaeoacremonium* species of the *P*. *minimum* clade were commonly isolated from wood of *V*. *vinifera*, various fruit trees and other deciduous trees, occasionally from soil or insects feeding on pruning wound sap of the infected grapevines, with one exception. Choi et al. (2011) reported a case of subcutaneous phaeohyphomycosis in Korea caused by *P*. *minimum* in a kidney transplant male patient as a consequence of a chronic renal failure caused by diabetes mellitus. Five years prior to the operation the patient had already a small nodule on his finger that gradually enlarged and was removed five years after the operation. The identity of the fungus isolated from the surgically removed mass was confirmed by ITS sequence via Blast search. The authors of the medical report [93] enabled us to study the ITS sequence of the pathogenic strain. Herewith we correct the identity of the Korean pathogenic strain as *P*. *iranianum* L. Mostert et al. [6], a species morphologically and phylogenetically closely related to *P*. *minimum*. Our conclusion is supported by phylogenetic study and a pairwise alignment of ITS sequences of the Korean strain and ex-type strain of *P*. *iranianum* CBS 101357 with 100% similarity. The original identification based on a Blast search was caused by inaccurate labelling of *Phaeoacremonium* sequences deposited in GenBank. The type strain of *P*. *iranianum* CBS 101357 and another strain of this species, CBS 101400, are still erroneously labelled as *P*. *minimum*. Nonetheless, this is the first case in which a species from the *P*. *minimum* clade was isolated from human tissue.

While comparing the ITS1 sequences of 13 members of the *P*. *parasiticum* subclade we observed that the ex-type strain of *P*. *parasiticum* CBS 860.73 had the shortest helices H2 and H3 in D3 domain. We compared this sequence with ITS sequences of eight other *P*. *parasiticum* strains available in GenBank. Their ITS sequences were identical except for the number of nucleotides forming canonical pairs in H2 and H3 duplexes. They can be distinguished into two groups. The ex-type strain CBS 860.73 and HD 337 [22, 94] isolated from subcutaneous lesion and foot abscess have H2 and H3 duplexes shorter by two bp. Strains of the other group with longer helices originated from different sources such as microfungal communities of garden-growing ants (strains CY 251, CY 123) [95], wood of *Prunus armeniaca* L. (CBS 121437) [15], wood of *Actinidia chinensis* Planch. (CBS 101007) [27], subcutaneous lesion on a kidney transplant patient (IFM 4924) [22], or respiratory tract of an immunocompromised patient with chemotherapy (PW 2367) [96]. The representation of strains in the second group is inconsistent regarding their ecology and pathogenicity to humans, however careful examination of growth characteristics and their morphology may reveal additional features that may unite them.

The main difference between the three *Phaeoacremonium* clades at the RNA structural level is obvious in the D3 and D4 domains of the ITS1 (Fig 5, Table 1). Species that grouped in each of the three clades share a unique topology of D3 characterised by the length of H2 and H3 duplexes of the 3WJ and phylogenetically conserved, unpaired nucleotides on the junction loop (see below). The D4 topology seemed also consistent within each clade and the number of predicted helices varied from one to two. Members of the *P*. *minimum* clade possess a constant string of CCCG unpaired nucleotides in L2 on 3WJ and two helices in the D4 domain. We distinguished three alternative patterns in D3 of the *P*. *parasiticum* clade corresponding to the recovered subclades. Species of the *P*. *parasiticum* and *P*. *krajdenii* subclades are characterised by a short sequence of GAA in L1 and a single G in L2. A short sequence of UCA nucleotides in L1 and a single G in L2 are typical of species of the *P*. *inflatipes* subclade, while the *P*. *sphinctrophorum* subclade is characterised by GA in L1 and CCCG in L2. In *P*. *sicilianum*, no unpaired nucleotides occur in the 3WJ loop. Two helices in D4 are a constant character of all species of the *P*. *parasiticum* clade, in *P*. *sicilianum* D4 consists of one long helix positioned near the 3’-end.

Whether the combination of molecular data with sexual morphological features, ecology and RNA structural characters may support a new classification of *Phaeoacremonium* at the generic or subgeneric levels will require additional study. Our hypothesis is corroborated by congruence of ascospore characters of the newly recognised sexual morph of *P*. *cinereum* and by the newly discovered grouping of *P*. *aquaticum* within the *P*. *parasiticum* clade based on ascospore shape and predicted 2D structure of ITS1. We do not know yet the phylogenetic placement of two *Phaeoacremonium* species known only as sexual morphs. Based on ascospore shape, *P*. *leptorrhynchum* can be preliminarily be assigned to the *P*. *minimum* clade, while *P*. *inconspicuum* should be a member of the *P*. *parasiticum* clade. However, we cannot rule out that their asexual morphs have already been described and the link between the two morphs awaits confirmation. The next research should entail further verification of the predictive value of sexual characters, while searching for strains with a heterothallic mating system in order to obtain ascomata in culture.

### Ascoma centrum in the Calosphaeriales and Togniniales

Although genera of both orders possess asci attached to the persistent ascogenous hyphae in predominantly spicate arrangement, the architecture of these formations is different. The ascoma centrum in the Calosphaeriales consists of septate paraphyses and ascogenous hyphae with minute, conspicuous cells, to which the asci are attached. In *Flabellascus* and *Togniniella* the ascogenous hyphae are conspicuously elongated with asci arising at various heights in spicate formation. In *Calosphaeria* and *Jattaea* the ascogenous hyphae are shorter, with asci in fascicles or palmate or short-spicate arrangements. Although in *Pleurostoma* the asci are also formed in fascicles or short-spicate formations, minute cells on ascogenous hyphae are inconspicuous and difficult to see in this genus.

In the Calosphaeriaceae, subglobose, pyriform or obovoid cells are formed on ascogenous hyphae from croziers in sympodial succession. These cells are persistent and can facilitate identification of the calosphaeriaceous fungi even in very old herbarium material. The origin of these cells is not clear. We discuss two possible scenarios, whether they are formed from croziers on ascogenous hyphae at the very beginning of the centrum formation and immature asci arise from them as an outgrowth, or they originate from the constricted ascus stipe. In the latter case, the basal part of the ascus stipe becomes constricted early in ontogeny, leaving a segment below the constriction attached to the ascogenous hypha, i.e. the future cell. The point of constriction is formed of the tightly folded outer and inner ascal wall and is always visible as a short, bristle-like appendage at the base of the attached or released ascus (e.g. Fig 11J). In Fig 13G of *J*. *tumidula* an immature ascus with clearly constricted base attached to a minute cell on the ascogenous hypha is shown.

In *Pleurostoma ootheca* the asci are oval with a short robust stipe, which is attached to the ascogenous hypha (Fig 15F, 15I and 15J). The deliberation of asci, their constriction at the base and formation of a bristle-like appendage is captured in Fig 15H; the appendages seem very flexible. A bristle formed by the inner ascal wall surrounded by remnants of the fractured outer wall is shown in Fig 15E. Our observations in the Pleurostomataceae would support the scenario, in which the cells on ascogenous hyphae are formed by constriction of the basal segment of the stipe.

In the Togniniaceae, the minute cells on ascogenous hyphae are lacking in all species of *Phaeoacremonium* with known sexual morphs. The asci are non-stipitate, obtuse at the base or with a very short stipe. They are formed directly on croziers on elongated and relatively wide, persistent ascogenous hyphae in sympodial succession. In most cases the whole ascus is deliberated, but sometimes the basal part of the ascus remains attached to the ascogenous hypha showing a slight fraying around the free edge, probably a result of a rupture in the ascal wall near the base. However, constriction of the stipe that we observed regularly in the Calosphaeriales has never been seen.

### Phylogenetic hypotheses of the Calosphaeriales and Togniniales according to the predicted RNA secondary structure of ITS

A close relationship between the Calosphaeriales and Togniniales was a premise for a comparison at the RNA structural level utilising their ITS sequences. Although Diaporthales is closely related to both orders, the ITS sequences of its members could not be aligned unless numerous gaps were introduced suggesting that many indels occurred during the evolution. The utility of predicted RNA secondary structure of ITS in systematics to define species complex groups was tested in various eukaryotic groups including the Ascomycota (e.g. [83, 97–109].

Based on our observation, the D3 domain of ITS1 provides the greatest variability at the RNA structural level and its topology characterises members of clades recovered in the Calosphaeriales and Togniniales. We confirm that the topology of helices of the three-way junction (3WJ) in D3 and distribution of unpaired nucleotides on the junction loop are phylogenetically conserved among species of genera of the Calosphaeriales and among species of the three major clades and several subclades of the Togniniales. They form a pattern that has a certain predictive value at the generic or subgeneric levels. Our results support the delimitation of five calosphaeriaceous genera at the RNA structural level and suggest a possible new classification of the Togniniales.

A similar 2D model of ITS1 comprising four domains was predicted for all members of the Calosphaeriaceae, Pleurostomataceae and Togniniaceae (Figs 3–5, Table 1). The D3 domain exhibits two different topologies. The primary sequences of D3 of species of the Calosphaeriaceae and Togniniaceae are folded into a three-way junction. The only exception to this pattern is represented by the Pleurostomataceae: the three-way junction is lacking and instead a single long helix occurs in all members of the family.

Three-way junctions serve as major architectural features that occur frequently in many RNA molecules including the ribosomal RNAs (e.g. [110–113] and hammerhead ribozymes [114, 115]. These structures are involved in a wide range of functional roles, including the self-cleaving catalytic domain of the hammerhead ribozyme [116] or the recognition of the binding pocket domain by purine riboswitches [117]. In the folded geometry of 3WJ, two helices are approximately coaxially stacked, while the third one is variously positioned to them, which results in recognition of the topological families A, B and C [118]. The helix-helix stacking provides a thermodynamic stability to the molecule as a whole and reduces the separation between loop regions within the junction, thus it is an essential feature of this motif [119, 120]. In previous computational studies it was shown that 3WJ of the types A and C exhibit specific flexibility that plays a role in important ribosomal processes [121, 122]. The role of unpaired nucleotides on the junction loop has been studied for example in biologically active 3WJs in the hammerhead ribozyme and in 5S rRNA. It was discovered that the unpaired nucleotides in the junction region of 5S rRNA are phylogenetically conserved [123].

We observed additional differences in 3WJ topology among genera of the Calosphaeriales and among species of the three major clades distinguished in *Phaeoacremonium* based on the classification of the 3WJ families and coaxial stacking of helices in the D3 domain. For this purpose we used two prediction programs, Junction Explorer [54] and Cartaj [55]. Based on the Junction Explorer prediction, all genera of the Calosphaeriales and Togniniales belong to the junction family C, while using the Cartaj prediction method, the 3WJ in D3 is predominantly of type A (for more details see Table 1). This tendency to shift between two different 3WJ families based on different computational methods has been reported by Schlick’s research group [54].

The predicted 2D models of the ITS2 molecule comprising a ring structure with four domains were more or less similar among members of the Calosphaeriales and Togniniales (Fig 6). The variable parts in each domain were predominantly hairpin loops differing in the number of nucleotides among genera. Of all domains only D4 was the most variable in ITS2 with significantly different length in the primary sequence among individual genera. Especially the ratio of canonical C = G and A-U pairs vs. U/G wobble pairs in D4 is an evidence of numerous substitutions in this part. In particular, the number of base pairs varies from three to thirteen in the Calosphaeriaceae, but is always two in the Pleurostomataceae and Togniniaceae.

## Supporting Information

S1 Table List of fungal names, isolate information and new sequences determined for this study and those retrieved from GenBank. GenBank accession numbers in bold were generated for this study. Strains: T = ex-holotype, Epi = ex-epitype, Is = ex-isotype.
(DOCX)
