# Supplementary material for: Phylogenetic Reconstruction of the Calosphaeriales and Togniniales Using Five Genes and Predicted RNA Secondary Structures of ITS, and Flabellascus tenuirostris gen. et sp. nov
Source: PLoS One. 2015 Dec 23;10(12):e0144616. doi: 10.1371/journal.pone.0144616 (PMC4689446; doi:10.1371/journal.pone.0144616)
Supplement: S1 Table — GenBank accession numbers in bold were generated for this study. Strains: T = ex-holotype, Epi = ex-epitype, Is = ex-isotype. (DOCX) [file pone.0144616.s001.docx]

**S1 Table. List of fungal names, isolate information and new sequences determined for this study and those retrieved from GenBank.**

| **Taxon** | **Source** | **Type** | **ITS** | **nuc28S** | **nuc18S** | **rpb2** | **β–tubulin** | **actin** |
| --- | --- | --- | --- | --- | --- | --- | --- | --- |
| **Calosphaeriales** |  |  |  |  |  |  |  |  |
| *Calosphaeria africana* | CBS 120870 | T | EU367444 | EU367454 | EU367460 | – | EU367464 | – |
| *Calosphaeria africana* | STE-U 6181 |  | EU367445 | EU367445 | EU367461 | – | EU367465 | – |
| *Calosphaeria pulchella* | CBS 115999 | T | EU367451 | AY761075 | AY761071 | GU180661 | **KT716476** | – |
| *Calosphaeria pulchella* | CCTU 316 |  | JX876610 | JX876611 | – | – | – | – |
| *Calosphaeria pulchella* | LM 06 |  | HM237298 | – | – | – | – | – |
| *Calosphaeria pulchella* | SS 07 |  | HM237297 | – | – | – | – | – |
| *Calosphaeria pulchella* | DC 04 |  | HM237299 | – | – | – | – | – |
| *Calosphaeria pulchella* | SM 05 |  | HM237300 | – | – | – | – | – |
| *Flabellascus tenuirostris* | CBS 138680 | T | **KT716466** | **KT716457** | – | **KT716475** | **KT716488** | – |
| *Flabellascus tenuirostris* | CBS 138690 |  | **KT716467** | **KT716458** | – | – | **KT716489** | – |
| *Flabellascus tenuirostris* | CBS 138692 |  | **KT716468** | **KT716459** | – | **KT716474** | **KT716483** | – |
| *Flabellascus tenuirostris* | CBS 139026 |  | **KT716469** | **KT716460** | – | – | **KT716484** | – |
| *Jattaea algeriensis* | CBS 120871 | T | EU367446 | EU367456 | EU367462 | HQ878603 | EU367466 | – |
| *Jattaea algeriensis* | STE-U 6400 |  | EU367448 | – | – | – | – | – |
| *Jattaea algeriensis* | STE-U 6399 |  | EU367448 | EU367457 | – | – | – | – |
| *Jattaea aphanospora* | PRM 934328 | T | HQ878588 | HQ878594 | – | – | **KT716477** | – |
| *Jattaea aurea* | CBS 140209 |  | **KT716462** | **KT716453** | **KT716447** | – | **KT716478** | – |
| *Jattaea discreta* | CBS 127681 |  | HQ878587 | HQ878593 | HQ878597 | – | **KT716479** | – |
| *Jattaea leucospermi* | CBS 119343 | T | EU552127 | EU552127 | – | – | – | – |
| *Jattaea mookgoponga* | CBS 120867 | T | HQ878589 | EU367458 | EU367463 | HQ878604 | EU367467 | – |
| *Jattaea mookgoponga* | STE-U 6401 |  | EU367450 | EU367459 | – | – | – | – |
| *Jattaea ribicola* | CBS 139779 | T | **KT716463** | **KT716454** | **KT716448** | – | **KT716480** | – |
| *Jattaea taediosa* | PRM 934412 | Epi | **KT716464** | **KT716455** | **KT716449** | – | **KT716481** | – |
| *Jattaea tumidula* | CBS 140208 | Epi | **KT716465** | **KT716456** | **KT716450** | – | **KT716482** | – |
| *Jattaea* sp. 1 | HNDC 06 |  | GU361954 | – | – | – | – | – |
| *Jattaea* sp. 2 | YNDC 23 |  | GU361945 | – | – | – | – | – |
| *Jattaea* sp. 2 | YNDC 19 |  | GU361941 | – | – | – | – | – |
| *Jattaea* sp. 3 | CBS 122684 |  | EU552160 | EU552160 | – | – | EU552167 | – |
| *Jattaea* sp. 3 | CMW 22119 |  | EU552159 | EU552159 | – | – | – | – |
| *Jattaea* sp. 4 | CBS 122685 |  | EU552161 | EU552161 | – | – | EU552168 | – |
| *Pleurostoma ootheca* | CBS 115329 | T | HQ878590 | AY761079 | AY761074 | HQ87860 | JX073272 | – |
| *Pleurostoma ochraceum* | CBS 131321 | T | JX073270 | JX073274 | JX073269 | – | JX073271 | – |
| *Pleurostoma repens* | CBS 294.39 | Is | AF083195 | AY761078 | AY761067 | – | JX073273 | – |
| *Pleurostoma richardsiae* | CBS 270.33 | T | AY179948 | AB364684 | AY761066 | HQ878607 | AY579334 | – |
| *Togniniella acerosa* | CBS 113726 | T | EU367452 | AY761077 | AY761072 | – | – | – |
| *Togniniella acerosa* | CBS 125298 |  | **KT716470** | **KT716461** | **KT716451** | GU180660 | **KT716485** | – |
| *Togniniella acerosa* | CBS 113648 |  | EU367453 | AY761076 | AY761073 | **KT716472** | **KT716486** | – |
| **Togniniales** |  |  |  |  |  |  |  |  |
| *Phaeoacremonium africanum* | STE-U 6177 | T | EU128052 | – | EU128060 | – | EU128100 | EU128142 |
| *Phaeoacremonium alvesii* | CBS 110034 | T | KF764558 | – | – | – | AY579301 | AY579234 |
| *Phaeoacremonium amygdalinum* | CBS 128570 | T | – | – | – | – | JN191307 | JN191303 |
| *Phaeoacremonium angustius* | CBS 114992 | T | **KU060813** | – | – | – | DQ173104 | DQ173127 |
| *Phaeoacremonium aquaticum* | IFRDCC 3035 | T | JQ797439 | – | – | – | – | – |
| *Phaeoacremonium amstelodamense* | CBS 110627 | T | KF764559 | – | – | – | AY579295 | AY579228 |
| *Phaeoacremonium armeniacum* | ICMP 17421 | T | EU770224 | – | – | – | EU596526 | EU595463 |
| *Phaeoacremonium argentinense* | CBS 777.83 | T | KF764569 | – | – | – | DQ173108 | DQ173135 |
| *Phaeoacremonium australiense* | CBS 120861 | T | EU128025 | – | – | – | EU128073 | AY579229 |
| *Phaeoacremonium austroafricanum* | CBS 112949 | T | KF764570 | – | – | – | DQ173099 | DQ173122 |
| *Phaeoacremonium canadense* | DAOM 242366 | T | KF764526 | – | – | – | KF764651 | KF764499 |
| *Phaeoacremonium cinereum* | CBS 123909 | T | KF764561 | – | – | – | FJ517161 | FJ517153 |
| *Phaeoacremonium cinereum* | CBS 138685 |  | **KT716471** | – | **KT716452** | **KT716473** | **KT716487** | – |
| *Phaeoacremonium croatiense* | CBS 123037 | T | EU863526 | – | – | – | EU863482 | EU863514 |
| *Phaeoacremonium fraxinopennsylvanicum* | CBS 101585 | T | AF295328 | AY761083 | – | – | KF764684 | DQ173137 |
| *Phaeoacremonium fraxinopennsylvanicum* | CBS 128920 |  | HQ878591 | HQ878595 | HQ878600 | HQ878609 | – | – |
| *Phaeoacremonium globosum* | ICMP 16988 | T | EU770229 | – | – | – | EU596525 | EU595466 |
| *Phaeoacremonium fuscum* | CBS 120856 | T | EU128050 | – | – | – | EU128098 | EU128141 |
| *Phaeoacremonium griseo–olivaceum* | STE-U 5966 | T | EU128049 | – | – | – | EU128097 | EU128139 |
| *Phaeoacremonium griseorubrum* | CBS 111657 | T | KF764562 | – | – | – | AY579294 | AY579227 |
| *Phaeoacremonium hispanicum* | CBS 123910 | T | KF764563 | – | – | – | FJ517164 | FJ517156 |
| *Phaeoacremonium hungaricum* | CBS 123036 | T | EU863525 | – | – | – | EU863483 | EU863515 |
| *Phaeoacremonium inflatipes* | CBS 391.71 | T | NR103566 | – | – | – | AF246805 | AY579259 |
| *Phaeoacremonium iranianum* | CBS 101357 | T | AY179942 | – | – | – | DQ173097 | DQ173120 |
| *Phaeoacremonium italicum* | CBS 137763 | T | KJ941005 | – | – | – | KJ534074 | KJ534046 |
| *Phaeoacremonium krajdenii* | CBS 109479 | T | – | – | – | – | AY579330 | AY579267 |
| *Phaeoacremonium krajdenii* | CBS 110368 |  | **KU060814** | – | – | – | AY579332 | AY579269 |
| *Phaeoacremonium krajdenii* | CBS 110118 |  | KF764571 | – | – | – | AY579324 | AY579261 |
| *Phaeoacremonium luteum* | CBS 137497 | T | **KU060815** | – | – | – | KF823800 | KF835406 |
| *Phaeoacremonium minimum* | CBS 246.91 | T | AB278174 | AB278174 | AB278174 | – | AF246811 | AY735497 |
| *Phaeoacremonium novae–zealandiae* | CBS 110156 | T | AY179945 | AY761081 | AY761069 | – | DQ173110 | DQ173139 |
| *Phaeoacremonium occidentale* | ICMP 17037 | T | EU770226 | – | – | – | EU596524 | EU595460 |
| *Phaeoacremonium pallidum* | CBS 120862 | T | EU128053 | – | – | – | EU128103 | EU128144 |
| *Phaeoacremonium parasiticum* | CBS 860.73 | T | U31841 | – | – | – | AF246803 | AY579253 |
| *Phaeoacremonium prunicolum* | CBS 120858 | T | EU128047 | – | – | – | EU128095 | EU128137 |
| *Phaeoacremonium roseum* | DAOM 242365 | T | KF764533 | – | – | – | KF764658 | KF764506 |
| *Phaeoacremonium rubrigenum* | CBS 498.94 | T | AB278173 | – | – | – | AF246802 | AY579238 |
| *Phaeoacremonium santali* | CBS 137498 | T | **KU060816** | – | – | – | KF823797 | KF835403 |
| *Phaeoacremonium scolyti* | CBS 113597 | T | KF764575 | – | – | – | AF246800 | AY579224 |
| *Phaeoacremonium sicilianum* | CBS 123034 | T | EU863524 | – | – | – | EU863488 | EU863520 |
| *Phaeoacremonium sphinctrophorum* | CBS 337.90 | T | **KU060817** | – | – | – | DQ173113 | DQ173142 |
| *Phaeoacremonium subulatum* | CBS 113584 | T | KF764565 | – | – | – | AY579298 | AY579231 |
| *Phaeoacremonium tardicrescens* | CBS 110573 | T | KF764566 | – | – | – | AY579300 | AY579233 |
| *Phaeoacremonium tuscanum* | CBS 123033 | T | EU863522 | – | – | – | EU863458 | EU863490 |
| *Phaeoacremonium venezuelense* | CBS 651.85 | T | KF764568 | – | – | – | AY579320 | AY579256 |
| *Phaeoacremonium vibratile* | CBS 117115 |  | – | – | – | – | DQ649063 | DQ649064 |
| *Phaeoacremonium viticola* | CBS 101738 | T | AF192391 | – | – | – | – | DQ173131 |
| *Phaeoacremonium theobromatis* | CBS 111586 | T | KF764567 | – | – | – | DQ173106 | DQ173132 |
| **Diaporthales** |  |  |  |  |  |  |  |  |
| *Gnomonia gnomon* | CBS 199.53 |  | – | AF408361 | DQ471019 | – | EU219148 | – |
| *Wuestneia molokaiensis* | CBS 114877 |  | – | AY720842 | – | – | AY579335 | AY579272 |

Legend

GenBank accession numbers in bold were generated for this study. Strains: T = ex–holotype, Epi = ex–epitype, Is = ex–isotype.
